# Widespread SARS-CoV-2 exposure in West Africa: findings from a 20,748-participant multi-country seroepidemiology study

**DOI:** 10.3389/fpubh.2026.1945355

**Published:** 2026-10-26

**Authors:** Aniefiok John Udoakang, Daniel Oduro-Mensah, Gloria Amegatcher, Onikepe Folarin, Isah Abubakar Aliyu, Irene Amoakoh Owusu, Francis Dzabeng, Alphonse Ouedraogo, Reuben Ayivor-Djanie, Jones Gyamfi, Moumouni Armel Sanou, Kesego Tapela, Manfreddy Binyet, Kyerewaa Akuamoah Boateng, Taiwo Gboluwaga Amole, Johnson Okolie, Baba Maiyaki Musa, Jamilu Abubakar Bala, Kazeem Akano, Ajibade Olufunke Olayemi, Olivia Achonduh-Atijegbe, Ajayi Nnennaya, Okoro Sylvanus Ihere, Azuogu Benedict, Okechukwu Ukpabi, Ojide Chiedozie Kingsley, Augustine Abu, Moyosoore Osoba, Nicholas Oyejide, Evans Agyapong Owusu, Bismark Adade, James Ekow Sampah, Samed Ishaq, George Agyei, Boateng Benjamin, William Oduro-Obeng, Janet Ogoe-Anderson, Nicholas Aloba Amekpordi, Safiatu Alhassan, David Afrifa, Bismark Akwasi Opoku, Oladele Oluwafemi Ayodeji, Chukwuyem Abejegah, Akinola Nelson Adedosu, Liasu Adeagbo Ahmed, Samuel S. Serme, Maurice Ouattara, Denise Hien, Nebié Issa, Rachael O. Boateng, Precious C. Opurum, Fatima O. Oyawoye, Dzidzor Yawa Ayeke, Michael Ofori, Nikiema Sindimbasba Achille, Bazoumana Sow, Lea Pare-Toe, Charles Guissou, Bismark Minna, Gifty Amponsah, Sylvester Donne Dassah, Sharif Alhassan Abdullahi, Nura Garba, Khadija Muhammad Bala, Rufai Umar Zubair, Mahfuz Muhammad Haddad, Lucas Amenga-Etego, Dominic S. Y. Amuzu, Collins M. Morang’a, Kwabena A. N. Sarpong, Patrick Williams Narkwa, William K Ampofo, Franklin Asiedu-Bekoe, Patrick Kuma-Aboagye, Kwabena O. Duedu, James Abugri, Mohamed Mutocheluh, Gideon Kofi Helegbe, Paul Armah Aryee, James Duah, Peter Yeboah, Yaw Bediako, Abdulai Diabate, Hadiza Shehu Galadanci, Sodiomon B. Sirima, Christian Happi, Gordon A. Awandare, Peter Kojo Quashie

**Affiliations:** 1 West African Centre for Cell Biology of Infectious Pathogens (WACCBIP), College of Basic and Applied Sciences, University of Ghana, Accra, Ghana; 2 Department of Biosciences and Biotechnology, University of Medical Sciences, Ondo, Nigeria; 3 Department of Biochemistry, Cell and Molecular Biology, University of Ghana, Accra, Ghana; 4 Department of Medical Laboratory Sciences, School of Basic and Allied Health Sciences, University of Ghana, Korle Bu, Ghana; 5 African Centre of Excellence for Genomics of Infectious Diseases (ACEGID), Redeemer's University, Ede, Nigeria; 6 Africa Center of Excellence for Population Health and Policy (ACEPHAP), Bayero University, Kano, Nigeria; 7 Groupe de Recherche Action en Santé (GRAS), Ouagadougou, Burkina Faso; 8 Department of Biomedical Sciences, School of Basic and Biomedical Sciences, University of Health and Allied Sciences, Ho, Ghana; 9 Department of Medical Laboratory Sciences, University of Health and Allied Sciences, Ho, Ghana; 10 Institut de Recherche en Sciences de la Santé (IRSS), Bobo-Dioulasso, Burkina Faso; 11 Department of Community Medicine, Bayero University Kano/ Aminu Kano Teaching Hospital, Kano, Nigeria; 12 Department of Medicine, College of Health Sciences, Bayero University, Kano, Nigeria; 13 Department of Medical Laboratory Sciences, College of Health Sciences, Bayero University, Kano, Nigeria; 14 St. Elizabeth Hospital, Hwidiem, Ghana; 15 St. Patrick Hospital, Offinso, Ghana; 16 SDA Hospital, Kwadaso, Ghana; 17 SDA Hospital, Obuasi, Ghana; 18 St. Peter’s Hospital, Jacobu, Ghana; 19 Methodist Faith Healing Hospital, Ankaase, Ghana; 20 Noguchi Memorial Institute for Medical Research, University of Ghana, Accra, Ghana; 21 Centre d’Excellence Africain en Innovations Biotechnologiques pour l’Élimination des, Maladies à Transmission Vectorielle (CEA/ITECH-MTV), Université Nazi BONI, Bobo-Dioulasso, Burkina Faso; 22 Department of Biochemistry and Forensic Sciences, School of Chemical and Biochemical Sciences, C. K. Tedam University of Technology and Applied Sciences, Navrongo, Ghana; 23 Department of Medical Microbiology and Parasitology, Faculty of Clinical Sciences, Bayero University, Kano, Nigeria; 24 Department of Nursing Sciences, Faculty of Allied Health Sciences, Bayero University, Kano, Nigeria; 25 Department of Clinical Microbiology, School of Medicine and Dentistry, Kwame Nkrumah University of Science and Technology, Kumasi, Ghana; 26 Ghana Health Service, Accra, Ghana; 27 School of Life and Health Sciences, Biirmingham City University, Birmingham, United Kingdom; 28 Department of Biochemistry and Molecular Medicine, School of Medicine, University for Development Studies, Tamale, Ghana; 29 Department of Nutritional Sciences, School of Allied Health Sciences, University for Development Studies, Tamale, Ghana; 30 Christian Health Association of Ghana (CHAG), Accra, Ghana; 31 Yemaachi Biotech, Accra, Ghana; 32 The Francis Crick Institute, London, United Kingdom; 33 Department of Gynecology and Obstetrics, College of Health Sciences, Bayero University, Kano, Nigeria

**Keywords:** Burkina Faso, COVID-19, Ghana, Nigeria, SARS-CoV-2, seroepidemiology, seroprevalence, West Africa

## Abstract

**Background:**

Africa seemed to have had low SARS-CoV-2 infection and COVID-19 morbidity and mortality. While several studies reported high SARS-CoV-2 seropositivity among healthy Africans, others hypothesized that this could have resulted from pre-existing cross-reactive antibodies. Our study tracks trends in seropositivity across three West African countries during the Delta- and Omicron-dominant pandemic periods using validated SARS-CoV-2 IgG/IgM rapid diagnostic tests (Prestige Diagnostics in Ghana and Burkina Faso; SD Biosensor in Nigeria).

**Methods:**

Cross-sectional studies were conducted in three West African countries (Burkina Faso, Ghana, and Nigeria), with study sites conveniently selected within each country. Using validated immunoassay kits with published validation procedures, SARS-CoV-2 antibodies were detected in peripheral blood during the Delta- and Omicron-dominant periods. Because we did not perform widespread viral sequencing on participant samples, we could not attribute seropositivity to a specific variant; instead, we classified sampling periods by the variant reported as dominant nationally/globally at the time of sample collection. Participants’ demographic and clinical data were obtained using a semi-structured questionnaire, and the primary outcome was the proportion of each population with SARS-CoV-2 antibodies.

**Results:**

Across the 3 countries, 20,748 participants were recruited, and SARS-CoV-2 IgG seropositivity ranged from 24.1 to 76.4%, with higher rates during the Omicron-dominant period. All sites showed low self-reported vaccination rates, suggesting that seropositivity may be primarily driven by natural infection rather than vaccination. While there was a significant positive association between seropositivity and age across all sites, the association with other demographic factors was country-dependent, and most participants expressed positive opinions of vaccination.

**Conclusion:**

This is one of the largest multi-country community-based SARS-CoV-2 seroepidemiology studies in Africa, showing widespread exposure to SARS-CoV-2 infection among our study population across three West African countries. These findings are consistent with a marked disconnect between high population exposure and low reported clinical burden, underscoring the need for strengthened community surveillance.

## 1 Introduction

The global spread of severe acute respiratory syndrome coronavirus 2 (SARS-CoV-2) in early 2020 raised concerns that fragile health systems in Africa would be overwhelmed (1). However, by September 2022, reported infection rates in Africa remained comparatively low at approximately 0.8%, compared with a global average of 7.5% (2, 3). Similarly, COVID-19-related mortality in Africa was estimated at 0.02%, which was lower than the global average of 0.08% at the time (3). These observations have been attributed to multiple factors, including younger population structure, underreporting of cases and deaths (4), and limited testing capacity in Africa (5). However, accumulating sero-epidemiological evidence suggests SARS-CoV-2 transmission in Africa has been wider than indicated by routine surveillance systems (6, 7). This shows widespread population exposure despite relatively low morbidity and mortality (8–10). This apparent disparity between infection rates and disease severity highlights a critical gap in understanding COVID-19 epidemiology and impact in Africa.

Sero-epidemiological studies are useful for estimating the true burden of infectious diseases, particularly in settings with limited testing capacity or scarce surveillance systems (11, 12). The detection of virus-specific antibodies provides a reliable means of assessing prior SARS-CoV-2 exposure at the population level, given the global naïve initial immunological status and the transient nature of acute infection (13, 14). Therefore, continued serological surveillance is important for understanding the dynamics of population immunity amid waning antibodies, vaccination, and the emergence of new viral variants.

In this study, parallel, multi-site sero-surveys were conducted across three West African countries (Burkina Faso, Ghana, and Nigeria) to estimate SARS-CoV-2 exposure in the region. Building on our previous work using validated lateral flow immunoassay-based rapid diagnostic tests (15), this study provides robust, regionally comparable estimates of SARS-CoV-2 seroprevalence contributing to a more accurate understanding of the impact of the pandemic in West Africa.

## 2 Materials and methods

### 2.1 Study design, setting, and sampling

Ethical approval was obtained from each country for the study, and written informed consent, including assent from minors (10–18 years) and consent from parents/guardians for participants below 18 years, was obtained before enrollment, questionnaire administration, and sample collection. The study followed the Helsinki Declaration guidelines.

This was a multi-site, community-based cross-sectional study using convenience sampling in three West African countries: Burkina Faso, Ghana, and Nigeria. Multiple sites within each country were conveniently selected at different times between June 2021 and June 2022. The sites were selected based on different socio-economic statuses and potential exposure to SARS-CoV-2, including shopping malls, markets, motor and lorry stations, religious settings (churches and mosques), and community centers (Table 1). The sampling periods were superimposed over the WHO reporting periods for easy appreciation and comparison (Supplementary Figure 1). As only limited viral sequencing was performed in Ghana on some participant samples (16), the sampling periods we refer to as the Delta-dominant period and Omicron-dominant period do not refer to a specific variant infecting participants but were instead used to classify the dominant variant reported nationally at the time of sample collection (Supplementary Figure 1). The study sites were Hauts-Bassins and Center in Burkina Faso; 13 of the 16 regions in Ghana; and in northern Nigeria, one state in the north (Kano), two states in the west (Osun and Ondo), and one state in the south (Ebonyi) (Supplementary Figure 2). Although participants were enrolled consecutively at all study sites, a subset of participants were members of higher educational institutions (staff and students) and personnel in COVID-19 testing facilities and hospitals.

**Table 1:** Demographics and vaccination history of study participants.

Participants (*N* = 20,748)	
Gender	
Male	8,145 (39.3%)
Female	12,603 (60.7%)
Age distribution (years)	
4–20	3,057 (14.7%)
21–40	10,951 (52.8%)
41–60	5,099 (24.6%)
60+	1,591 (7.7%)
Missing	50 (0.2%)
Location (Country)	
Burkina Faso	2,458 (11.9%)
Ghana	9,981 (48.1%)
Nigeria	8,309 (40.0%)
Sampling sites	
Shopping Malls	1,157 (5.6%)
Markets	7,554 (36.4%)
Institutions	2,475 (11.9%)
Community	4,699 (22.7%)
Religious settings	1,454 (7.0%)
Health Centre/Hospital	3,409 (16.4%)
COVID-19 vaccination status	
Yes, Dose 1	2,906 (14.0)
Yes, Dose 1 & 2	2,440 (11.8)
No	15,402 (74.2)
Vaccine types	
AstraZeneca	3,905 (73%)
Sputnik V	3 (0.1%)
Johnson & Johnson	547 (10.2%)
Pfizer	207 (3.9%)
Moderna	503 (9.4%)
Others (Sinopharm)	7 (0.1%)
Do not Know	174 (3.3%)

Participants were approached by any of the COVID-19 surveillance team members. After the study objectives and protocols were described to them, those who accepted and gave informed consent to participate in the study were recruited. Adults signed informed consent, and assent was obtained for participants who were less than 18 years old, with consent from their parents/guardian. Recruitment sites were not identical between the two sampling periods, and the distribution of participants across site types differed between periods within each country (Table 2).

**Table 2:** Background characteristics of participants by country from June 2021 – June 2022 (*N* = 20,748).

Characteristics	Burkina Faso			Ghana			Nigeria		
	Total	Delta	Omicron	Total	Delta	Omicron	Total	Delta	Omicron
	*n* (%)	*n* (%)	*n* (%)	*n* (%)	*n* (%)	*n* (%)	*n* (%)	*n* (%)	*n* (%)
Recruitment sites									
Shopping malls				509 (5.1)	509 (8.5)	0 (0.0)	648 (7.8)	365 (6.6)	283 (10.1)
Markets	1952 (79.4)	1,468 (74.4)	484 (100)	3,170 (31.8)	1,610 (26.9)	1,560 (39.0)	2,432 (29.3)	1,550 (28.1)	882 (31.5)
Institutions	206 (8.4)	206 (10.4)	0 (0.0)	1,604 (16.1)	872 (14.6)	732 (18.3)	665 (8.0)	653 (11.9)	12 (0.4)
Community/Households				2,266 (22.7)	795 (13.3)	1,471 (36.8)	2,433 (29.3)	1,397 (25.4)	1,036 (37.0)
Religious settings	294 (12.0)	294 (14.9)	0 (0.0)	953 (9.5)	810 (13.5)	143 (3.6)	207 (2.5)	0 (0.0)	207 (7.4)
Health Centre/Hospital	6 (0.2)	6 (0.3)	0 (0.0)	1,479 (14.8)	1,385 (23.2)	94 (2.4)	1924 (23.2)	1,542 (28.0)	382 (13.6)
Total	2,458 (100)	1974 (100)	484 (100)	9,981 (100)	5,981 (100)	4,000 (100)	8,309 (100)	5,507 (100)	2,802 (100)
Age group (in years)									
4–20	276 (11.2)	244 (12.4)	32 (6.6)	1,530 (15.3)	836 (14.0)	694 (17.3)	1,251 (15.1)	858 (15.6)	393 (14.0)
21–40	1,328 (54.0)	1,087 (55.1)	241 (49.8)	5,057 (50.7)	3,316 (55.4)	1741 (43.5)	4,566 (55.0)	2,928 (53.2)	1,638 (58.5)
41–60	668 (27.2)	499 (25.3)	169 (34.9)	2,502 (25.1)	1,318 (22.0)	1,184 (29.6)	1929 (23.2)	1,304 (23.7)	625 (22.3)
60+	183 (7.4)	141 (7.1)	42 (8.7)	854 (8.6)	493 (8.2)	361 (9.0)	554 (6.7)	416 (7.6)	138 (4.9)
Missing	3 (0.1)	3 (0.2)	0 (0.0)	38 (0.4)	18 (0.3)	20 (0.5)	9 (0.1)	1 (0.0)	8 (0.3)
Total	2,458 (100)	1974 (100)	484 (100)	9,981 (100)	5,981 (100)	4,000 (100)	8,309 (100)	5,507 (100)	2,802 (100)
Gender									
Male	1,129 (45.9)	955 (48.4)	174 (36.0)	3,764 (37.7)	2,275 (38.0)	1,489 (37.2)	3,252 (39.1)	2,215 (40.2)	1,037 (37.0)
Female	1,329 (54.1)	1,019 (51.6)	310 (64.0)	6,217 (62.3)	3,706 (62.0)	2,511 (62.8)	5,057 (60.9)	3,292 (59.8)	1765 (63.0)
Total	2,458 (100)	1974 (100)	484 (100)	9,981 (100)	5,981 (100)	4,000 (100)	8,309 (100)	5,507 (100)	2,802 (100)
Education level									
None/Basic	1,381 (56.2)	1,048 (53.1)	333 (68.8)	1964 (19.7)	1,064 (17.8)	900 (22.5)	1733 (20.9)	1,044 (19.0)	689 (24.6)
Secondary	725 (29.5)	610 (30.9)	115 (23.8)	5,029 (50.4)	2,662 (44.5)	2,367 (59.2)	4,304 (51.8)	2,768 (50.3)	1,536 (54.8)
Undergraduate	228 (9.3)	208 (10.5)	20 (4.1)	2,671 (26.8)	2012 (33.6)	659 (16.5)	2068 (24.9)	1,546 (28.1)	522 (18.6)
Postgraduate	124 (5.0)	108 (5.5)	16 (3.3)	220 (2.2)	157 (2.6)	63 (1.6)	196 (2.4)	148 (2.7)	48 (1.7)
Missing				97 (1.0)	86 (1.4)	11 (0.3)	8 (0.1)	1 (0.0)	7 (0.2)
Total	2,458 (100)	1974 (100)	484 (100)	9,981 (100)	5,981 (100)	4,000 (100)	8,309 (100)	5,507 (100)	2,802 (100)
Monthly income									
Low	1,418 (57.7)	1,219 (61.8)	199 (41.1)	7,507 (75.2)	4,192 (70.1)	3,315 (82.9)	5,911 (71.1)	3,976 (72.2)	1935 (69.1)
Middle	728 (29.6)	511 (25.9)	217 (44.8)	2000 (20)	1,405 (23.5)	595 (14.9)	1,102 (13.3)	313 (5.7)	789 (28.2)
High	311 (12.7)	243 (12.3)	68 (14)	125 (1.3)	78 (1.3)	47 (1.2)	1,291 (15.5)	1,217 (22.1)	74 (2.6)
Missing	1 (0.0)	1 (0.1)	0 (0.0)	349 (3.5)	306 (5.1)	43 (1.1)	5 (0.1)	1 (0.0)	4 (0.1)
Total	2,458 (100)	1974 (100)	484 (100)	9,981 (100)	5,981 (100)	4,000 (100)	8,309 (100)	5,507 (100)	2,802 (100)
Employment type									
Formal	317 (12.9)	292 (14.8)	25 (5.2)	1950 (19.5)	1,401 (23.4)	549 (13.7)	1,443 (17.4)	1,066 (19.4)	377 (13.5)
Informal	1,611 (65.5)	1,214 (61.5)	397 (82)	4,697 (47.1)	2,526 (42.2)	2,171 (54.3)	4,559 (54.9)	2,900 (52.7)	1,659 (59.2)
Student	388 (15.8)	356 (18.0)	32 (6.6)	2086 (20.9)	1,178 (19.7)	908 (22.7)	1,115 (13.4)	872 (15.8)	243 (8.7)
Unemployed/Retired	118 (4.8)	90 (4.6)	28 (5.8)	1,096 (11.0)	744 (12.4)	352 (8.8)	1,183 (14.2)	669 (12.1)	514 (18.3)
Missing	24 (1.0)	22 (1.1)	2 (0.4)	152 (1.5)	132 (2.2)	20 (0.5)	9 (0.1)	0 (0.0)	9 (0.3)
Total	2,458 (100)	1974 (100)	484 (100)	9,981 (100)	5,981 (100)	4,000 (100)	8,309 (100)	5,507 (100)	2,802 (100)

### 2.2 Participant testing

The Prestige Diagnostics IgG/IgM COVID-19 rapid diagnostic test (RDT) antibody card kit (Lot No. 3120200506) and SureStatus COVID-19 antigen card test (Lot No. 97D0321S) were used for SARS-CoV-2 antibody and antigen detection, respectively. The manufacturer-reported sensitivity and specificity of the Prestige Diagnostics assay were 91.8 and 96.4% for IgG, and 95.7 and 97.3% for IgM, respectively (17). An independent clinical evaluation of the SureStatus antigen test reported an overall sensitivity of 82.4% and specificity of 98.5% (18).

In Nigeria, the SD Biosensor COVID-19 IgG/IgM Combo Rapid diagnostic test kits [58329F2AC/Sub:1 Buffer: STEB1021683], SD Biosensor COVID-19 antigen rapid diagnostic test kits [Lot Number 58328A1AC], and Abbott Panbio COVID-19 antigen rapid test device [Lot number 41ADG178A, Diluent 41ADDG179] (Abbott Rapid Diagnostics Jena GmbH, Germany) were used. For the SD Biosensor STANDARD Q COVID-19 IgM/IgG Duo used in Nigeria, an independent evaluation by the U. S. National Cancer Institute and the Food and Drug Administration reported a combined sensitivity of 76.7% (95% CI: 59.1–88.2%) and a specificity of 98.8% (93.3–99.8%), with an IgG sensitivity of 73.3% (55.6–85.8%) and an IgM sensitivity of 53.3% (36.1–69.8%) (38). The antigen test kits were used for same-day referrals of potentially infectious participants; antigen results are not included in this seroprevalence analysis.

Participants with temperatures greater than 38 °C were protocol-excluded from analysis (not recruitment) because active fever could cause elevated IgM levels and increase the risk of cross-reactivity linked with malaria endemicity (19). That being said, fever was rare in our study participants. After testing, participants were informed of their exposure status and, where necessary, advised to seek further testing. Participants who tested positive for IgM or were antigen-positive were referred to COVID-19 test and treatment facilities for further monitoring. We use ‘seropositivity’ in our reporting to denote the detection of SARS-CoV-2-specific antibodies via rapid diagnostic testing, which serves as a proxy for prior exposure or infection rather than virologically confirmed infection.

A semi-structured questionnaire was used to obtain demographic, health history, previous COVID-19 status, and vaccination information. Data was collected by trained interviewers using the World Bank Survey solution suite, an online application with integrated quality checks. Participants’ information was de-identified before data analysis. Questionnaire data, including reasons for vaccine hesitancy, were analyzed descriptively (frequencies and percentages); no formal qualitative analysis was undertaken.

### 2.3 Data analysis

The data were cleaned by checking for completeness, duplication, and consistency, and were analyzed with Stata 16 (StataCorp, College Station, Texas, USA) and R/RStudio. Descriptive statistics were performed, and log-binomial regression models were used to assess the factors associated with seropositivity. Log-binomial regression models were fitted to estimate prevalence ratios (PRs) and 95% confidence intervals for factors associated with SARS-CoV-2 seropositivity. Age group, sex, recruitment site, education level, monthly income, and employment type were specified *a priori* for consideration in multivariable analyses due to their epidemiological relevance. Additional variables associated with seropositivity at *p* < 0.25 in univariable analyses were considered during model building. Multivariable models were developed using a combination of epidemiological judgment and model parsimony, and resulting associations should be interpreted as exploratory. Model adequacy was assessed through convergence diagnostics and comparison of Akaike Information Criterion (AIC) values among candidate models. Prevalence ratios with 95% confidence intervals were computed, and statistical significance was inferred for *p*-values below 0.05. As this is a cross-sectional study with exposure and outcome measured concurrently, prevalence ratios and associated estimates are interpreted as measures of association rather than prediction or temporally ordered risk.

## 3 Results

### 3.1 Demographic profile of study participants

A total of 20,748 participants were recruited into the study over two sampling periods: Burkina Faso (2458), Ghana (9981), and Nigeria (8309) (Figure 1). The demographic profile of all the study participants by country is presented in Tables 1, 2. The first sampled period (June 2021–Nov 2021) corresponded to the period during which Delta was the globally/nationally dominant circulating variant, while the second period (Dec 2021 - June 2022) corresponded to the period during and after the global infection wave during which Omicron and Omicron+ subvariants were dominant. There were more female participants (60.7%) than males; the modal age range was between 21 and 40 years, and most participants were recruited from markets (Table 1). In Burkina Faso, most participants were recruited from religious settings (79.4%), while they were almost evenly recruited across all sites in Ghana and Nigeria (Table 2). A similar age distribution was observed among the participants, with the 21–40 years age group more represented in Burkina Faso (54%) and Nigeria (55%). Also, participants above 60 years old were least represented (Burkina Faso 7.4%; Ghana 8.6%; Nigeria 6.7%) (Table 2). While most participants (56.2%) in Burkina Faso had basic or no education, about half of the participants in Ghana (50.4%) and Nigeria (51.8%) had up to secondary education (Table 2). While between 47.1 and 65.5% of the participants worked in informal settings and 12.9 to 19.5% in formal settings, 4.8 to 14.2% had no employment.

**Figure 1:**
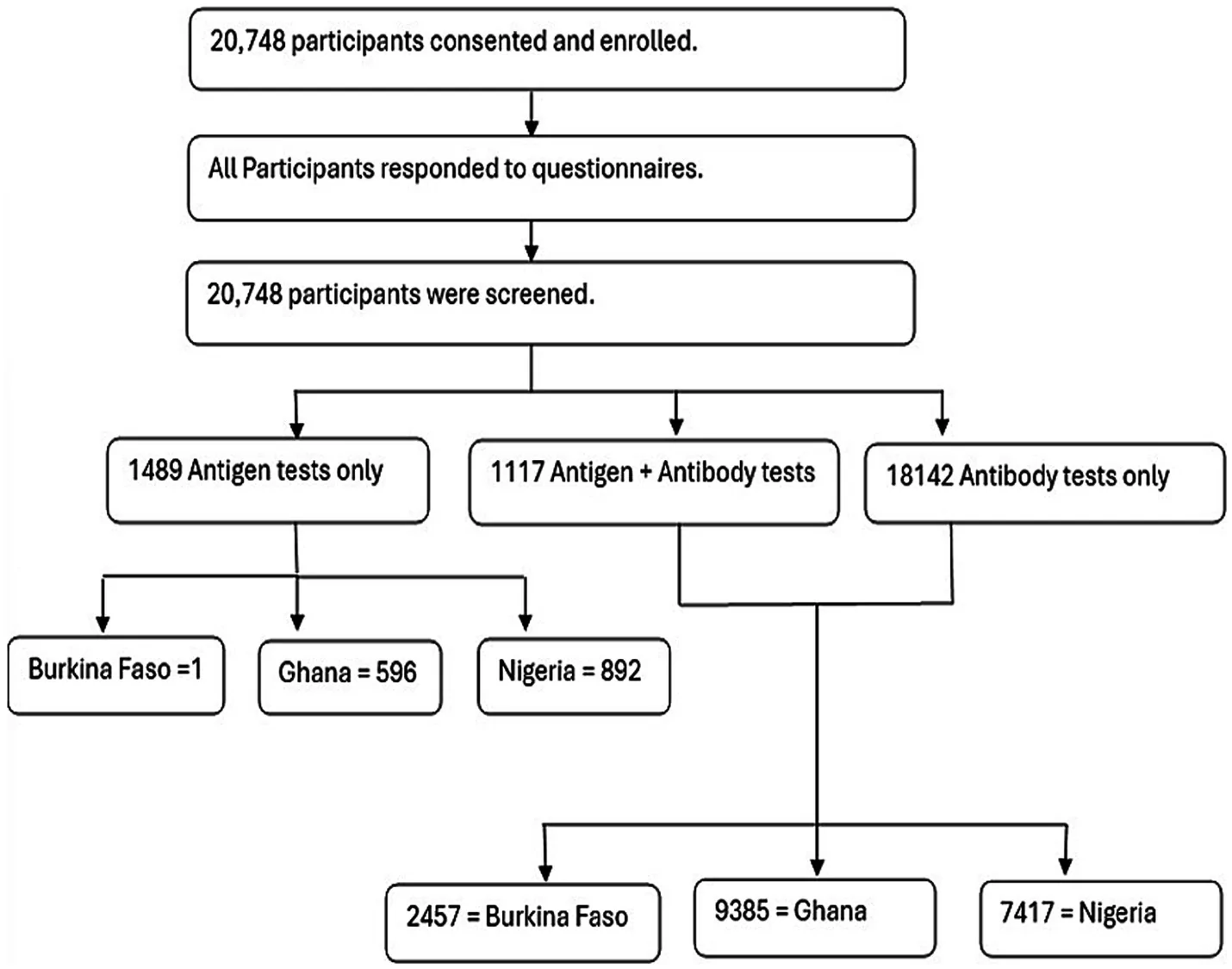
Flow diagram of the 20,748 participants enrolled and screened, showing the distribution of antigen and antibody testing across Burkina Faso, Ghana, and Nigeria.

### 3.2 COVID-19 sero-epidemiology across study sites

Overall seropositivity for both sampling periods across all three countries was 52.8% [52.1–53.5], with 3.9% [3.6–4.2] IgM seropositivity and 51.5% [50.8–52.2] IgG seropositivity (Supplementary Table 1).

#### 3.2.1 Burkina Faso

In Burkina Faso, overall seropositivity was 24.9% [23.0–26.8] during the Delta-dominant period, which was lower than the 51.0% [46.4–55.5] (overall; IgG 52.9% [48.4–57.3]) recorded during the Omicron-dominant period (Figure 2). When considering only the unvaccinated, the exposure was 22.7% in the Delta-dominant period compared to 43.5% in the Omicron-dominant period (Supplementary Figure 3). During the Delta-dominant period, the highest seropositivity was recorded among participants recruited at religious settings (43.5%), with the lowest among those at health centers (16.7%) (Supplementary Table 2a). Seropositivity among the 60 + age group was highest (32.6 and 61.9% in the Delta- and Omicron-dominant periods, respectively). Higher seropositivity rates were found among undergraduates and postgraduates in the Delta-dominant period (31.9 and 32.4%, respectively), which changed to include the less educated (54.1 and 48.7%) in the Omicron-dominant period. The highest seropositivity rates in both periods were recorded among those in the highest income (31.3%, 63.2%) group and those who worked in formal settings (30.5 and 64.0%), although most participants had low income and worked in the informal sector (Supplementary Table 2a).

**Figure 2:**
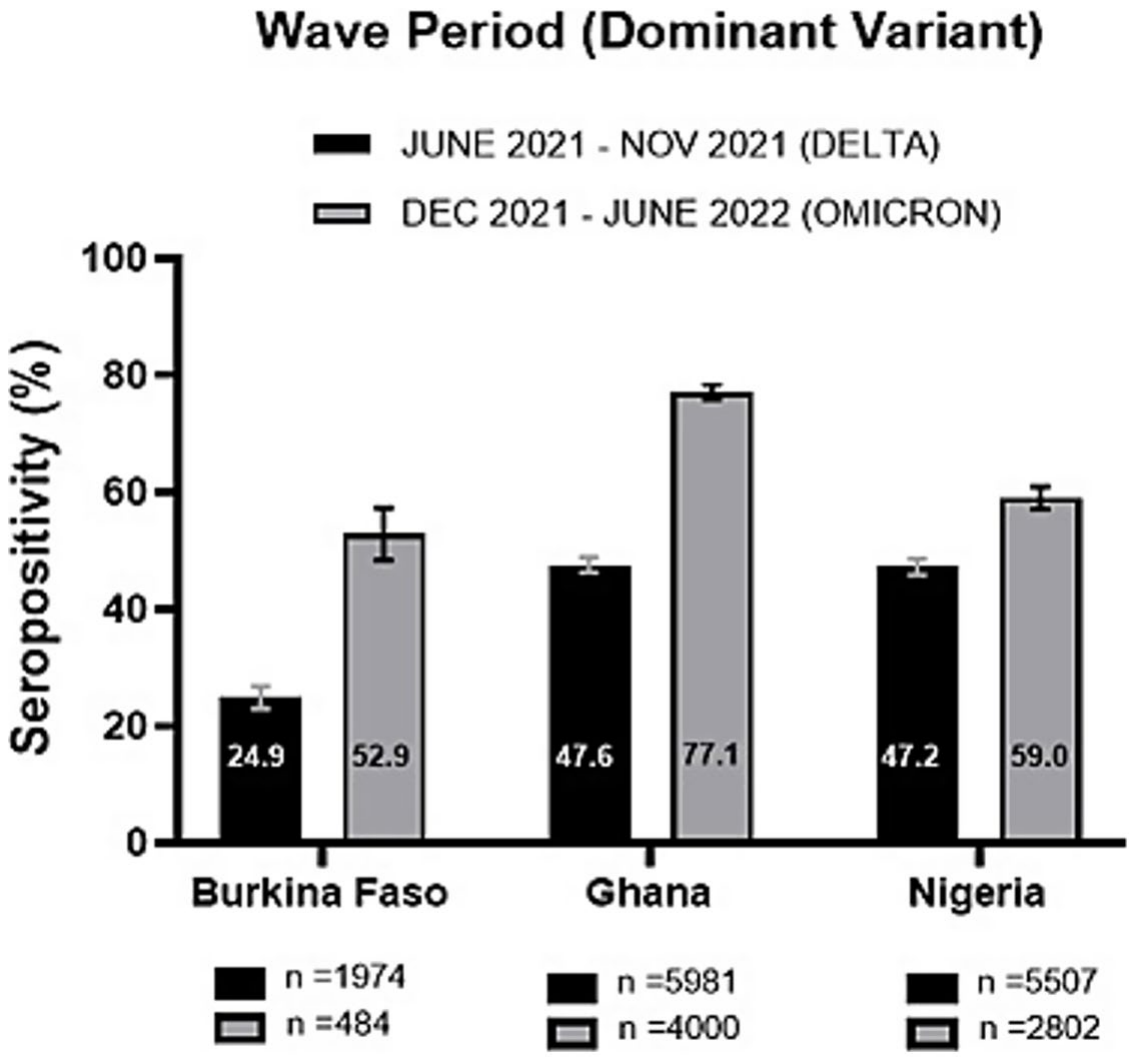
Comparison of SARS-CoV-2 seropositivity (IgM and IgG) across Burkina Faso, Ghana, and Nigeria during the Delta- and Omicron-dominant periods. Bars represent the percentage of participants who were seropositive, with sample sizes indicated below each bar.

#### 3.2.2 Ghana

Ghana recorded a lower seropositivity of 47.6% [46.3–48.9] during the Delta-dominant period compared to 77.1% [75.8–78.4] during the Omicron-dominant period (Figure 2); among unvaccinated individuals, 35.2 and 57.8% were exposed during the Delta- and Omicron-dominant periods, respectively (Supplementary Figure 3). Seropositivity varied from 39.3 to 53.8% during the Delta-dominant period and from 60.0 to 83.1% in the Omicron-dominant period, with the highest prevalence among participants recruited from markets and religious settings. Interestingly, participants recruited at healthcare facilities recorded the lowest seropositivity during both periods (Supplementary Table 2b). During both periods, seropositivity was highest among postgraduates (57.8 and 81%), though the ranges were smaller during the Omicron-dominant period. Also, participants with high incomes had higher seropositivity rates in both periods, with the same trend as those employed in the formal sector during the Delta-dominant period (48.5%). Seropositivity within all the groups remained high (73.7- 79.9%), though there were higher rates among the students during the Omicron-dominant period (Supplementary Table 2b).

#### 3.2.3 Nigeria

In Nigeria, there was an increase from 47.2% [45.8–48.8] during the Delta-dominant period compared to 59.0% [57.1–61.0] during the Omicron-dominant period (Figure 2), with exposure levels of 38.5 and 42.3% in the unvaccinated during the Delta- and Omicron-dominant periods, respectively (Supplementary Figure 3). The highest seroprevalence was recorded in shopping malls (67.7%) during the Delta-dominant period and in markets (70.2%) during the Omicron-dominant period (Supplementary Table 2c). During the Delta-dominant period, there was higher seropositivity among those aged 41–60 (49.4%), while the highest seropositivity was observed among those aged 60 + during the Omicron-dominant period (Supplementary Tables 2a–2c). At the first recruitment period, the postgraduate education (60.1%), middle-income (68.8%), and unemployed/retired (60.6%) groups recorded the highest seropositivity rates in their respective categories. Secondary education (61.7%), middle-income status (62.3%), and informal employment (63.2%) had the highest rates during period 2 (Supplementary Table 2c).

### 3.3 Factors associated with IgG positivity over the sampling periods

#### 3.3.1 Recruitment site

Relative to being sampled in an institution, participants recruited in religious settings in Ghana were more likely to be exposed (1.2[1.1–1.3]; *p* < 0.001), while those sampled in shopping malls in Nigeria had higher exposure (1.3[1.2–1.5]; *p* < 0.001) during the Delta-dominant period. Interestingly, in Burkina Faso, sampling in Markets/Motor parks seemed to be associated with lower seropositivity (0.6[0.5–0.7]; *p* < 0.001) during the Delta-dominant period (Supplementary Table 3a). However, during the Omicron-dominant period, as seroprevalence was rising, people had a similar likelihood of being seropositive in any recruitment site in Ghana and Nigeria (Supplementary Table 3b).

#### 3.3.2 Age

The likelihood of seropositivity appeared to increase with participants’ age during the Delta-dominant period in Burkina Faso and Ghana (1.7[1.2–2.5]; *p* = 0.002 and 1.3[1.1–1.5]; *p* < 0.001) (Supplementary Table 3a), while this association was significant only in Ghana and Nigeria during the Omicron-dominant period (1.2[1.1–1.2]; *p* < 0.001 and 1.4[1.2–1.6]; *p* < 0.001) (Supplementary Table 3b).

#### 3.3.3 Sex

Over the two sampling periods in Burkina Faso, there was no significant association between gender (Delta: 0.8[0.7–1.0], *p* = 0.010; Omicron: 1.1[0.9–1.3]; *p* = 0.476); however, there was a slight likelihood of seropositivity among Females in Ghana (Delta: 1.1[1.1–1.2]; *p* < 0.001; Omicron: 1.1[1.1–1.2]; *p* < 0.001) and in Nigeria (Delta: 0.86[0.8–0.92]; *p* < 0.001, Omicron: 0.86[0.8–0.92]; *p* < 0.001). These data suggest that the likelihood of seropositivity was not too different between genders (Supplementary Tables 3a, 3b).

#### 3.3.4 Educational level

During the Delta-dominant period, postgraduates in the three countries were more likely to be seropositive (Burkina Faso 1.4[1.1–1.9], *p* = 0.014; Ghana 1.3[1.1–1.5], *p* = 0.004; Nigeria 1.5 [1.2–1.7], *p* < 0.001) than those with no formal or basic education. This association was with both undergraduates and postgraduates in Burkina Faso and Nigeria (Supplementary Table 3a). However, during the subsequent waves, there was no difference in associations between seropositivity across all educational level groups (Supplementary Table 3b).

#### 3.3.5 Income

During the Delta-dominant period, those earning higher monthly income were more likely to be seropositive in all three countries (Burkina Faso 1.4[1.1–1.7]; *p* = 0.004, Ghana 1.4[1.1–1.6]; *p* = 0.001, Nigeria 1.1[1.0–1.2]; *p* = 0.009) (Supplementary Table 3a). However, the association was only observed within this group in Burkina Faso (1.4[1.1–1.8]; *p* = 0.007) during the Omicron-dominant period. This association seemed not to be very important as the pandemic spread across the countries (Supplementary Tables 3a, 3b).

#### 3.3.6 Employment

Relative to working in the formal sector, where middle- and higher-income individuals are, those in the informal sector seemed to be less likely to be seropositive in Burkina Faso (0.8[0.6–1.0]; *p* = 0.017) and Nigeria (0.8[0.8–0.9]; *p* < 0.001) during the Delta-dominant period (Supplementary Table 3a). However, as the pandemic evolved, these associations attenuated for most employment statuses: Informal (Burkina Faso 0.8[0.6–1.1]; *p* = 0.193 Ghana 0.96[0.9–1.0]; *p* = 0.093); Student (Ghana 1.0[0.95–1.1]; *p* = 0.643 Nigeria 1.0[0.8–1.1]; *p* = 0.678); unemployed (Burkina Faso 0.8[0.5–1.3]; *p* = 0.442 Ghana 1.0[0.9–1.1]; *p* = 0.808 Nigeria 1.0[0.8–1.1]; *p* = 0.722) (Supplementary Table 3b).

### 3.4 Association of clinical characteristics with seropositivity

In Ghana and Burkina Faso, self-reported clinical characteristics were not associated with exposure; no significant association between symptoms 4 weeks earlier and seropositivity was observed (Supplementary Table 4). In Nigeria, there was a significant association between seropositivity and most known COVID-19 symptoms, including shortness of breath (*p* < 0.001), cough (*p* < 0.001), anosmia (*p* < 0.001), headache (*p* < 0.001), sore throat (*p* < 0.001), and runny nose (*p* < 0.001).

The most common chronic condition reported in each country was heart disease, recorded in 9.6%, 8.3%, and 4.2% of participants from Burkina Faso, Ghana, and Nigeria, respectively, which was positively associated with seropositivity in Ghana (*p* < 0.001) and Nigeria (*p* = 0.006). Of the other major chronic diseases queried, only diabetes showed any association with seropositivity in Ghana (*p* = 0.019) alone (Supplementary Table 5).

### 3.5 Association of participant hospitalization or COVID-19 testing with seropositivity

Only in Ghana (*p* < 0.001) was there an association observed between seropositivity and hospitalization in the 12 weeks preceding the survey, and COVID-19 PCR testing outcomes. However, there was no association of seropositivity with COVID-19 PCR testing outcomes or duration since the COVID-19 test. This supports the idea of massive undetected asymptomatic SARS-CoV-2 spread (Supplementary Table 6).

### 3.6 Association of COVID-19 vaccination and opinion of vaccination with seropositivity

A majority (74.23%) of all participants were unvaccinated for COVID-19 at the time of the survey (Table 1). By country, Burkina Faso recorded the highest proportion (92.1%) of unvaccinated, while Ghana recorded the least (59.9%) (Supplementary Table 7). In each country, proportions of individuals who had received at least one vaccine dose increased with time (Figures 3A,B), and the AstraZeneca vaccine (73%) was the most common vaccine received (Table 1; Supplementary Table 7).

**Figure 3:**
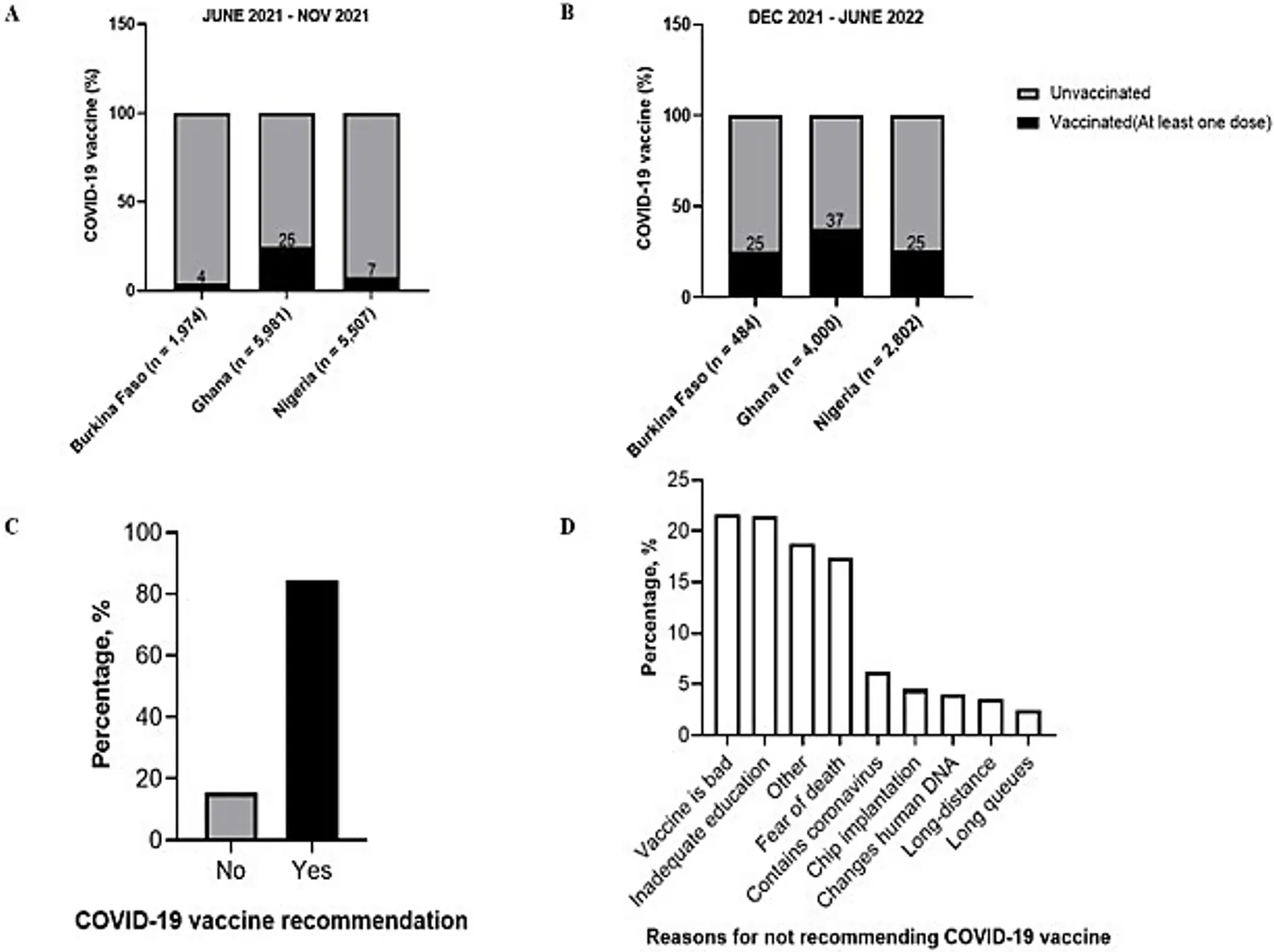
COVID-19 vaccination status and vaccine recommendation among study participants in all 3 study areas. Proportion of vaccinated and unvaccinated participants during the Delta variant-dominant sampling period (June–November 2021) **(A)** and Omicron variant-dominant sampling period (December 2021 – June 2022) **(B)**. Graph showing proportion of participants who would recommend COVID-19 vaccines to others **(C)** and reasons for not recommending **(D)**.

Most study participants (84.4%) indicated that they would recommend the COVID-19 vaccine to others (Figure 3C), and for those who would not recommend the vaccine, the top three reasons for not recommending vaccination were: (1) the ‘vaccine is bad’ (21.6%), (2) little or no vaccine education (21.4%), and (3) taking the vaccine may kill them (17.4%) (Figure 3D).

The willingness to recommend vaccines to others (Supplementary Table 8) was differentially associated with age (*p* < 0.001), high educational level (*p* < 0.009), and Employment (*p* < 0.001) in Ghana and Nigeria, Gender (*p* = 0.002) in Burkina Faso, and Monthly income in Burkina Faso (*p* = 0.009) and Ghana (*p* = 0.004).

## 4 Discussion

Sero-epidemiological studies have provided essential insights into the spread of diseases, including COVID-19. We previously conducted the first population-based survey of COVID-19 in Africa, spanning mid-2020 to early 2021 (D614G to Alpha waves) (15) along with large-scale household-based surveillance across Greater Accra, Ghana (20). This focus on Ghana was limited in its ability to predict trends across West Africa. In our study, seropositivity was higher than in a previous Ghanaian ‘Alpha wave’ study (20). Also, in a multi-city serosurvey in Ghana, post-stratified seroprevalence rates of about 53.3% were reported in Accra and 84.7% in Tamale by early 2022, revealing an estimated infection-to-clinical-case ratio of 85:1 and 49:1, respectively. This massive decoupling between population exposure and clinical presentations is mirrored across the sub-region (21).

This study is one of the largest SARS-CoV-2 seroprevalence studies in Africa and used an extensive questionnaire to collect key data on factors that could affect exposure and seropositivity, such as recent clinical factors, occupations, and socioeconomic status. Conducted in three West African countries, Burkina Faso, Ghana, and Nigeria, this wider geographical representation allowed us to examine reported clinical burden alongside seropositivity across the sub-region.

The most consequential SARS-CoV-2 nationally dominant variants during our sampling windows were Delta (June–November 2021), Omicron (December 2021–February 2022), and Omicron-plus (March 2022 - date) (Figure 2), though individual infections were not sequence-confirmed in this study. Our seroprevalence data covering the same period showed that seropositivity mirrored Africa’s infection waves, particularly in Ghana (Supplementary Figure 4). Seropositivity in the separately sampled Omicron-period populations was higher than in the Delta-period populations in all three countries (absolute differences of approximately 26, 29 and 12 percentage points in Burkina Faso, Ghana and Nigeria, respectively; Figure 2). Because these were repeated cross-sectional samples whose composition, recruitment settings, and prevailing restrictions differed between periods, and because limited viral sequencing was performed, these differences could not directly be attributed to the greater transmissibility of Omicron, although they are consistent with the wider spread reported globally during the Omicron-dominant period. It is worth noting that because the Omicron-dominant period corresponded to relaxed lockdown protocols and easing of government-related COVID-19 restrictions in these countries, this might have contributed to the higher exposure among the population. Intriguingly, Ghana’s government lifted most COVID-19-related restrictions in April 2020 (22) after the country recorded low clinically significant COVID-19 cases. However, Burkina Faso and Nigeria eased their lockdown restrictions around July 2020 (23). Our data, showing high SARS-CoV-2 seropositivity, particularly at the time restrictions were eased, suggest cumulative exposure to SARS-CoV-2 in our sampled population across the sub-region. Given that individuals showed antibodies without a history of recent clinical disease, this finding suggests widespread prior exposure to SARS-CoV-2 in the study population. This corroborates a nationwide study across 12 states in Nigeria, which showed a high community seroprevalence of 78.9% despite concurrently low hospitalization rates (24).

Being 60 years and above demonstrated the strongest association with seropositivity, particularly during the Omicron-dominant period. Previously, age was a critical determinant of the clinical course of COVID-19: older people around this age were likely to have severe disease, require prolonged treatment, and, in such a population, infection was associated with higher complications and mortality (25–28). In this study, age was significantly associated with seropositivity across the three countries, consistent with more widespread previous exposure, but without an overt increase in clinically significant COVID-19, particularly among the older adults. This observation is in line with the literature discussing the curiously low COVID-19 in West Africa, though severe cases and deaths from the disease were not accounted for in this study (29, 30).

Sampling from institutions, shopping malls, and religious centers had the highest occurrence of IgM positivity. This is consistent with expected respiratory dynamics within enclosed spaces, although we did not directly measure the environmental viral loads. Sampling from market/motor/lorry parks was associated negatively with seropositivity during the Delta-dominant period in Burkina Faso and further supports the suggestion that congregating in open spaces did not enhance transmission during the Delta-dominant period. Although there was very little clinical COVID-19 during the Omicron-dominant period, IgG seropositivity proportions at all sites were similar to or higher than in the Delta-dominant period, suggesting potentially higher exposures. During the Delta-dominant period, sampling in shopping malls, where an individual was likely to encounter high-income individuals, or in the case of Nigeria, middle-income individuals, was associated with higher seropositivity than among low-income individuals across the study sites. This apparent relationship between income status, sampling at a shopping mall, and seropositivity may reflect distinct mobility patterns of the group during lockdown periods, although they may not be the definitively identified primary exposure site. The positive association of seropositivity with an increasing educational level in Ghana and Nigeria provides further evidence of this trend, potentially reflecting a higher likelihood of formal employment among the relatively educated individuals. Immunocompromised status, exemplified by HIV infection, did not show a significant association with SARS-CoV-2 seropositivity. However, fear of stigmatization, due to mistrust, especially in the kind of response that individuals could receive, may have affected this answer, since only 29 people (0.019% of respondents) reported HIV-positive status. The case of mistrust was also expressed in a study carried out in the US among people living with HIV (PLWH) who had COVID-19 (31).

In Burkina Faso and Ghana, seropositivity was primarily driven by natural infection rather than vaccination, since the test kits detected anti-SARS-CoV-2 nucleocapsid IgG and IgM for actual SARS-CoV-2 infection, not the spike protein expressed in the vaccines utilized. Tests in Nigeria did not distinguish between Spike and Nucleocapsid antibodies, but the effect on seropositivity was expected to be minimal since Nigerian participants reported the second-lowest vaccination rates (8.4%) over the two periods (Supplementary Table 7). This is further supported by the persistence of high seropositivity among unvaccinated participants across all sites (Supplementary Figure 3), indicating that the observed antibody prevalence predominantly reflects natural infection rather than vaccine-derived immunity.

Despite the low reported clinical burden, high SARS-CoV-2 seropositivity was observed among the predominantly non-febrile study participants. The observed disconnect between high SARS-CoV-2 exposure and low reported clinical severity in our cohort is striking. While most participants (59.9–92.1%) were not vaccinated, a significant proportion (up to 76.4%) had been exposed to SARS-CoV-2 and reported less severe or no symptoms at the time of recruitment. However, because our study measured binding antibodies (IgG/IgM) rather than neutralizing capacity, and because we excluded febrile individuals at the time of sampling, we cannot conclude that this population was uniformly protected from severe disease; rather, our data document widespread exposure with an observed low clinical burden. This pattern does not diminish the importance of mass vaccination policy, which remains essential for protecting vulnerable individuals, preventing health-system collapse, and ending the global pandemic (32–35). The factors underlying the high proportion of asymptomatic or low-severity presentation observed in this study across West Africa are not yet known, although a combination of host, environmental, and epidemiological factors, including endemic malaria, may be a contributing factor (36, 37). Our cross-sectional design cannot disentangle these hypotheses. In the case of SARS-CoV-2, as in the case of other outbreaks, it is impossible to predict individuals or regions with immunity/protection. Vaccines protect the vulnerable and remain a critical part of disease control. It is thus encouraging that most participants (84.4%) appreciate the importance of vaccines. On the other hand, there are misconceptions tied to anti-vaxxer misinformation campaigns that should be dispelled in this population. An improved and committed approach to communicating science to the public is needed.

Major limitations of this study were the use of convenience sampling and difficulty in harmonizing the different incomes, lifestyles, and public spaces across the three countries. The convenience sampling could limit the generalizability of our study estimates. In Burkina Faso, men were the predominant respondents due to the social structure. This and other country-level variations may have affected the concordance across the sites for certain factors such as income, gender, and education.

Likewise, another key limitation of our study is the reliance on different serological assays across study sites. The Prestige Diagnostics RDT, which targets nucleocapsid (N) antibodies, was used in Ghana and Burkina Faso, while the SD Biosensor assay, used in Nigeria, captures other profiles, including spike (S) responses. Thus, as regional vaccination rates progressed, the Nigerian cohort data could have reflected a combination of vaccine-induced immunity and natural infection, while the Ghanaian and Burkina Faso cohorts could reflect natural exposure alone, even despite the extremely low reported vaccination rates. Due to these inter-assay differences and antigen divergence, direct cross-country comparisons should be interpreted with caution, most likely as relative trends rather than absolute differences. Also, because the sampling strategy was localized and varied between countries, these regional seroprevalence patterns reflect the specific attributes of the sampled clusters. It is important to note that viral sequencing was not performed across all sites and, when performed (16), was not on all samples; therefore, seropositivity could not be directly linked to a specific variant in this case but rather to nationally reported circulating variants-some of which were from our study cohorts. Because the mix of recruitment sites differed between the Delta- and Omicron-dominant periods (for example, the Omicron-period sample in Burkina Faso was recruited entirely in markets), differences in seropositivity between periods may partly reflect differences in the populations sampled rather than temporal change in exposure alone. Additionally, we assessed symptom history for 4 weeks preceding the survey; some participants may have experienced symptoms more than 4 weeks before the survey but did not require hospitalization. Therefore, the observed recent asymptomatic infection in our participants should not be interpreted as a direct measure of asymptomatic infections accumulated across previous waves.

### 4.1 Statistical limitations

The regression analyses did not explicitly account for clustering of participants within recruitment sites, regions, or countries. Individuals sampled from the same location may share environmental, socioeconomic, behavioral, or epidemiological characteristics, which could reduce the independence of observations. Consequently, confidence intervals may be narrower than would be obtained using clustered or hierarchical modeling approaches. Therefore, associations identified in the regression analyses should be interpreted as exploratory.

A large number of statistical comparisons were conducted across demographic, socioeconomic, clinical and behavioral variables, and no formal adjustment for multiple testing was performed. Consequently, some statistically significant findings may reflect chance associations (Type I error). To mitigate this risk, emphasis was placed on the magnitude, direction and precision of estimated prevalence ratios and their 95% confidence intervals rather than on statistical significance alone. Therefore, observed associations should be interpreted as exploratory and warrant confirmation in future studies.

## 5 Conclusion

The SARS-CoV-2 seroprevalence data reported here suggest that: 1. The substantially higher seropositivity observed in the sampled populations than indicated by routinely reported cases is consistent with substantial under-ascertainment of SARS-CoV-2 infection by symptom-based surveillance. 2. Among our participants who were asymptomatic at the time of sample collection and 4 weeks prior, the proportion of seropositivity was significant and progressed with the pandemic. 3. The high seropositivity observed in SARS-CoV-2 sero-surveys could be linked to recent SARS-CoV-2 exposure, rather than being solely attributed to pre-pandemic cross-reactive antibodies. Seropositivity, particularly in Burkina Faso and Ghana, where nucleocapsid-specific assays exclude vaccine-derived antibodies and further supported by persistently high seropositivity among the unvaccinated (Supplementary Figure 3), which predominantly reflects natural infection rather than vaccination. Our data from seropositivity testing suggest that persons without recent symptoms may still be exposed to such respiratory viruses that can spread and maintain transmission in the population, indicating that reliance on hospital attendance alone is likely not a good method to estimate the prevalence of respiratory infections due to high levels of seropositivity in asymptomatic individuals. While our findings are consistent with a population-level disconnect between high SARS-CoV-2 exposure and low clinical burden in West Africa, the extent to which this reflects biological protection, demographic structure, or surveillance gaps remains to be determined through prospective, longitudinal studies.

## Data Availability

The original contributions presented in the study are included in the article/Supplementary material, further inquiries can be directed to the corresponding authors.
