# Supplementary material for: Widespread SARS-CoV-2 exposure in West Africa: findings from a 20,748-participant multi-country seroepidemiology study
Source: Front Public Health. 2026 Oct 26;14:1945355. doi: 10.3389/fpubh.2026.1945355 (PMC13752853; doi:10.3389/fpubh.2026.1945355)
Supplement: Supplementary file 1 [file Supplementary_file_1.DOCX]

**Supplementary Table1**: Summary of Seropositivity by region within each country from June 2021 to June 2022

|  |  |  |  | **IgG Positivity** |  | **IgM Positivity** |  | **Overall Positivity** |
| --- | --- | --- | --- | --- | --- | --- | --- | --- |
|  |  |  |  |  |  |  |  |  |
|  |  |  |  | ***^% (95% CI)^*** |  | ***^% (95% CI)^*** |  | ***^% (95% CI)^*** |
|  | ***All countries/sites*** | ***^20,748^*** |  | ***51.5 (50.8 - 52.2)*** |  | ***3.9*** *(****3.6 - 4.2)*** |  | ***52.8 (52.1 – 53.5)*** |
| **Country** | **REGION/STATE** |  |  |  |  |  |  |  |
| **BURKINA FASO** | BOBO DIOULASSO | 967 |  | 32.6 (29.7 - 35.6) |  | 2.4 (1.59 -3.6) |  | 33.5 (30.6 - 36.5) |
|  | OUAGADOUGOU | 1491 |  | 27.4 (25.2 - 29.7) |  | 3.0 (2.2 - 4.0) |  | 28.4 (26.2 - 30.7) |
|  | **Overall** | **2,458** |  | **29.4 (27.7 - 31.3)** |  | **2.7 (2.2 - 3.5)** |  | **30.4 (28.6 - 32.3)** |
|  | AHAFO | 348 |  | 85.1 (80.9 - 88.4) |  | 8.6 (6.1 - 12.1) |  | 85.9 (81.9 - 89.2) |
|  | ASHANTI | 1,849 |  | 60.5 (58.1 - 62.8) |  | 3.9 (3.1 - 5.0) |  | 61.3 (58.9 - 63.6) |
|  | BONO | 287 |  | 45.9 (39.2 - 52.7) |  | 14.0 (9.9 - 19.4) |  | 51.7 (44.9 - 58.4) |
|  | BONO EAST | 262 |  | 51.5 (45.1 - 58.0) |  | 7.9 (5.1 - 12.2) |  | 55.9 (49.4 - 62.3) |
|  | CENTRAL | 805 |  | 54.1 (50.4 - 57.8) |  | 3.3 (2.2 - 4.9) |  | 54.9 (51.2 - 58.6) |
|  | EASTERN | 778 |  | 68.7 (65.2 - 72.1) |  | 8.1 (6.3 - 10.3) |  | 71.0 (67.5 - 74.2) |
| **GHANA** | GREATER ACCRA | 2,679 |  | 61.7 (59.9 - 63.5) |  | 3.0 (2.4 - 3.7) |  | 62.2 (60.4 - 64.0) |
|  | NORTHERN | 446 |  | 34.5 (30.2 - 39.1) |  | 1.1 (0.5 - 2.7) |  | 35.0 (30.7 - 39.6) |
|  | SAVANNAH | 363 |  | 81.6 (77.0 - 85.4) |  | 7.3 (5.0 - 10.6) |  | 84.6 (80.3 - 88.1) |
|  | UPPER EAST | 799 |  | 47.4 (44.0 - 50.9) |  | 4.0 (2.9 - 5.6) |  | 49.6 (46.1 - 53.0) |
|  | UPPER WEST | 334 |  | 59.0 (53.6 - 64.1) |  | 3.0 (1.6 - 5.5) |  | 59.6 (54.2 - 64.7) |
|  | VOLTA | 646 |  | 44.9 (41.0 - 48.9) |  | 3.8 (2.5 - 5.7) |  | 45.7 (41.8 - 49.7) |
|  | WESTERN | 385 |  | 63.5 (58.5 - 68.3) |  | 2.2 (1.1 - 4.3) |  | 64.6 (59.6 - 69.3) |
|  | **Overall** | **9,981** |  | **58.8 (57.8 - 59.8)** |  | **4.3 (3.9 - 5.0)** |  | **60.0 (59.0 - 61.0)** |
|  | EBONYI STATE | 1,406 |  | 45.3 (42.7 - 47.9) |  | 4.1 (3.2 - 5.3) |  | 45.9 (43.4 - 48.6) |
| **NIGERIA** | KANO STATE | 3,117 |  | 55.3 (53.2 - 57.3) |  | 4.8 (4.0 - 5.8) |  | 58.7 (56.7 - 60.8) |
|  | ONDO STATE | 1,898 |  | 58.5 (56.3 - 60.7) |  | 3.7 (3.0 - 4.7) |  | 59.0 (56.7 - 61.2) |
|  | OSUN | 1,888 |  | 36.7 (34.6 - 38.9) |  | 2.3 (1.7 - 3.1) |  | 37.9 (35.7 - 40.1) |
|  | **Overall** | **8,309** |  | **49.5 (48.4 - 50.6)** |  | **3.8 (3.4 - 4.2)** |  | **51.1 (49.9 - 52.2)** |

N = number of participants tested; percentages use the regional N as the denominator. The All countries row pools all 20,748 participants

**Supplementary Table 2a**: Background Characteristics by Seropositivity in Burkina Faso (JUNE 2021 – MAY 2022)

|  | **(Delta-dominant period)^a^** | | | | | | |  | | **(Omicron-dominant period)^b^** | | | | | |
| --- | --- | --- | --- | --- | --- | --- | --- | --- | --- | --- | --- | --- | --- | --- | --- |
|  |  | **IgG** |  | **IgM** |  | **Overall** | |  | |  | **IgG** |  | **IgM** |  | **Overall** |
|  | **Total** | **Positivity** |  | **Positivity** |  | **Positivity** | |  | | **Total** | **Positivity** |  | **Positivity** |  | **Positivity** |
|  | **N** | **n (%)** |  | **n (%)** |  | **n (%)** | |  | | **N** | **n (%)** |  | **n (%)** |  | **n (%)** |
| **Recruitment Sites** |  |  |  |  |  |  | |  | |  |  |  |  |  |  |
| Shopping Malls |  |  |  |  |  |  | |  | |  |  |  |  |  |  |
| Markets | 1468 | 285 (19.4) |  | 19 (1.3) |  | 291 (19.8) | | |  | 484 | 256 (52.9) |  | 26 (5.4) |  | 247 (51.0) |
| Institutions | 205 | 67 (32.7) |  | 11 (5.4) |  | 71 (34.6) | |  | |  |  |  |  |  |  |
| Community |  |  |  |  |  |  | |  | |  |  |  |  |  |  |
| Religious settings | 294 | 123 (41.8) |  | 11 (3.7) |  | 128 (43.5) | | |  |  |  |  |  |  |  |
| Health Centre/Hospital | 6 | 1 (16.7) |  | 0 (0.0) |  | 1 (16.7) | |  | |  |  |  |  |  |  |
| **Total** | **1973** | **476 (24.1)** |  | **41 (2.1)** |  | **491 (24.9)** | | |  | **484** | **256 (52.9)** |  | **26 (5.4)** |  | **247 (51.0)** |
|  |  |  |  |  |  |  | |  | |  |  |  |  |  |  |
| **Age group (in years)** |  |  |  |  |  |  | |  | |  |  |  |  |  |  |
| 4-20 | 244 | 46 (18.9) |  | 8 (3.3) |  | 48 (19.7) | |  | | 32 | 13 (40.6) |  | 3 (9.4) |  | 12 (37.5) |
| 21-40 | 1086 | 252 (23.2) |  | 23 (2.1) |  | 262 (24.1) | | |  | 241 | 115 (47.7) |  | 12 (5.0) |  | 110 (45.6) |
| 41-60 | 499 | 132 (26.5) |  | 8 (1.6) |  | 135 (27.1) | | |  | 169 | 102 (60.4) |  | 7 (4.1) |  | 99 (58.6) |
| 60+ | 141 | 46 (32.6) |  | 2 (1.4) |  | 46 (32.6) | |  | | 42 | 26 (61.9) |  | 4 (9.5) |  | 26 (61.9) |
| Missing | 3 | 0 (0.0) |  | 0 (0.0) |  | 0 (0.0) | |  | |  |  |  |  |  |  |
| **Total** | **1973** | **476 (24.1)** |  | **41 (2.1)** |  | **491 (24.9)** | | |  | **484** | **256 (52.9)** |  | **26 (5.4)** |  | **247 (51.0)** |
|  |  |  |  |  |  |  | |  | |  |  |  |  |  |  |
| **Gender** |  |  |  |  |  |  | |  | |  |  |  |  |  |  |
| Male | 955 | 255 (26.7) |  | 23 (2.4) |  | 263 (27.5) | | |  | 174 | 89 (51.1) |  | 16 (9.2) |  | 85 (48.9) |
| Female | 1018 | 221 (21.7) |  | 18 (1.8) |  | 228 (22.4) | | |  | 310 | 167 (53.9) |  | 10 (3.2) |  | 162 (52.3) |
| **Total** | **1973** | **476 (24.1)** |  | **41 (2.1)** |  | **491 (24.9)** | | |  | **484** | **256 (52.9)** |  | **26 (5.4)** |  | **247 (51.0)** |
|  |  |  |  |  |  |  | |  | |  |  |  |  |  |  |
| **Education level** |  |  |  |  |  |  | |  | |  |  |  |  |  |  |
| None/Basic | 1048 | 235 (22.4) |  | 17 (1.6) |  | 244 (23.3) | | |  | 333 | 185 (55.6) |  | 16 (4.8) |  | 180 (54.1) |
| Secondary | 610 | 142 (23.3) |  | 11 (1.8) |  | 146 (23.9) | | |  | 115 | 59 (51.3) |  | 8 (7.0) |  | 56 (48.7) |
| Undergraduate | 207 | 64 (30.9) |  | 9 (4.3) |  | 66 (31.9) | |  | | 20 | 8 (40.0) |  | 1 (5.0) |  | 7 (35.0) |
| Postgraduate | 108 | 35 (32.4) |  | 4 (3.7) |  | 35 (32.4) | |  | | 16 | 4 (25.0) |  | 1 (6.3) |  | 4 (25.0) |
| Missing |  |  |  |  |  |  | |  | |  |  |  |  |  |  |
| **Total** | **1973** | **476 (24.1)** |  | **41 (2.1)** |  | **491(24.9)** |  | | | **484** | **256 (52.9)** |  | **26 (5.4)** |  | **247 (51.0)** |
|  |  |  |  |  |  |  | |  | |  |  |  |  |  |  |
| **Monthly income** |  |  |  |  |  |  | |  | |  |  |  |  |  |  |
| Low | 1218 | 274 (22.5) |  | 26 (2.1) |  | 285 (23.4) | | |  | 199 | 94 (47.2) |  | 6 (3.0) |  | 91 (45.7) |
| Middle | 511 | 126 (24.7) |  | 7 (1.4) |  | 129 (25.2) | | |  | 217 | 116 (53.5) |  | 14 (6.5) |  | 113 (52.1) |
| High | 243 | 75 (30.9) |  | 8 (3.3) |  | 76 (31.3) | |  | | 68 | 46 (67.6) |  | 6 (8.8) |  | 43 (63.2) |
| Missing | 1 | 1 (100) |  | 0 (0.0) |  | 1 (100) | |  | |  |  |  |  |  |  |
| **Total** | **1973** | **476 (24.1)** |  | **41 (2.1)** |  | **491 (24.9)** | | |  | **484** | **256 (52.9)** |  | **26 (5.4)** |  | **247 (51.0)** |
| **Employment type** |  |  |  |  |  |  | |  | |  |  |  |  |  |  |
| Formal | 292 | 87 (29.8) |  | 11 (3.8) |  | 89 (30.5) | |  | | 25 | 18 (72.0) |  | 3 (12.0) |  | 16 (64.0) |
| Informal | 1214 | 282 (23.2) |  | 18 (1.5) |  | 291 (24) | |  | | 397 | 213 (53.7) |  | 21 (5.3) |  | 207 (52.1) |
| Student | 355 | 80 (22.5) |  | 11 (3.1) |  | 84 (23.7) | |  | | 32 | 9 (28.1) |  | 1 (3.1) |  | 8 (25.0) |
| Unemployed/Retired | 90 | 17 (18.9) |  | 1 (1.1) |  | 17 (18.9) | |  | | 28 | 15 (53.6) |  | 1 (3.6) |  | 15 (53.6) |
| Missing | 22 | 10 (45.5) |  | 0 (0.0) |  | 10 (45.5) | |  | | 2 | 1 (50.0) |  | 0 (0.0) |  | 1 (50.0) |
| **Total** | **1973** | **476 (24.1)** |  | **41 (2.1)** |  | **491(24.9)** |  | | | **484** | **256 (52.9)** |  | **26 (5.4)** |  | **247 (51.0)** |
|  |  |  |  |  |  |  | |  | |  |  |  |  |  |  |

^a^ June 2021 – November 2021 (Delta-dominant period); ^b^ December 2021 – May 2022 (Omicron-dominant period). Percentages within each period use participants with available serology results in each category as the denominator; category totals reflect the analytic subset with non-missing data and may differ from other tables.

**Supplementary Table 2b**: Background Characteristics by Seropositivity in Ghana (JUNE 2021 – JUNE 2022)

|  | **(Delta-dominant period)^a^** | | | | | | | |  | **(Omicron-dominant period)^b^** | | | | | |
| --- | --- | --- | --- | --- | --- | --- | --- | --- | --- | --- | --- | --- | --- | --- | --- |
|  |  | **IgG** | |  | | **IgM** |  | **Overall** |  |  | **IgG** |  | **IgM** |  | **Overall** |
|  | **Total** | **Positivity** | |  | | **Positivity** |  | **Positivity** |  | **Total** | **Positivity** |  | **Positivity** |  | **Positivity** |
|  | **N** | **n (%)** | |  | | **n (%)** |  | **n(%)** |  | **N** | **n (%)** |  | **n (%)** |  | **n(%)** |
| **Study site/location** |  |  | |  | |  |  |  |  |  |  |  |  |  |  |
| Shopping Mall | 509 | 262 (51.5) | | |  | 31 (6.1) |  | 273 (53.6) |  |  |  |  |  |  |  |
| Markets | 1610 | 794 (49.3) | | |  | 57 (3.5) |  | 816 (50.7) |  | 1560 | 1177 (75.4) |  | 59 (3.8) |  | 1184 (75.9) |
| Institutions | 856 | 375 (43.8) | | |  | 31 (3.6) |  | 386 (45.1) |  | 732 | 603 (82.4) |  | 16 (2.2) |  | 608 (83.1) |
| Community | 794 | 325 (40.9) | | |  | 35 (4.4) |  | 339 (42.7) |  | 1471 | 1112 (75.6) |  | 76 (5.2) |  | 1122 (76.3) |
| Religious settings | 808 | 424 (52.5) | | |  | 45 (5.6) |  | 435 (53.8) |  | 143 | 103 (72.0) |  | 9 (6.3) |  | 107 (74.8) |
| Health Centre/Hospital | 852 | 317 (37.2) | | |  | 42 (4.9) |  | 335 (39.3) |  | 50 | 29 (58.0) |  | 2 (4.0) |  | 30 (60.0) |
| **Total** | 5429 | 2497 (46.0) | |  | | 241 (4.4) |  | 2584 (47.6) |  | 3956 | 3024 (76.4) |  | 162 (4.1) |  | 3051 (77.1) |
|  |  |  | |  | |  |  |  |  |  |  |  |  |  |  |
| **Age group (in years)** |  |  | |  | |  |  |  |  |  |  |  |  |  |  |
| 4-20 | 757 | 319 (42.1) | | |  | 40 (5.3) |  | 334 (44.1) |  | 691 | 503 (72.8) |  | 23 (3.3) |  | 512 (74.1) |
| 21-40 | 3061 | 1355 (44.3) | | |  | 103 (3.4) |  | 1397 (45.6) |  | 1716 | 1280 (74.6) |  | 59 (3.4) |  | 1294 (75.4) |
| 41-60 | 1200 | 599 (49.9) | | |  | 71 (5.9) |  | 625 (52.1) |  | 1170 | 928 (79.3) |  | 50 (4.3) |  | 930 (79.5) |
| 60+ | 395 | 215 (54.4) | | |  | 27 (6.8) |  | 219 (55.4) |  | 359 | 303 (84.4) |  | 30 (8.4) |  | 305 (85.0) |
| Missing | 16 | 9 (56.3) | |  | | 0 (0.0) |  | 9 (56.3) |  | 20 | 10 (50.0) |  | 0 (0.0) |  | 10 (50.0) |
| **Total** | 5429 | 2497 (46) | |  | | 241 (4.4) |  | 2584 (47.6) |  | 3956 | 3024 (76.4) |  | 162 (4.1) |  | 3051 (77.1) |
|  |  |  | |  | |  |  |  |  |  |  |  |  |  |  |
| **Gender** |  |  | |  | |  |  |  |  |  |  |  |  |  |  |
| Male | 2048 | 874 (42.7) | | |  | 100 (4.9) |  | 903 (44.1) |  | 1471 | 1046 (71.1) |  | 75 (5.1) |  | 1061 (72.1) |
| Female | 3381 | 1623 (48.0) | |  | | 141 (4.2) |  | 1681 (49.7) |  | 2485 | 1978 (79.6) |  | 87 (3.5) |  | 1990 (80.1) |
| **Total** | 5429 | 2497 (46.0) | |  | | 241 (4.4) |  | 2584 (47.6) |  | 3956 | 3024 (76.4) |  | 162 (4.1) |  | 3051 (77.1) |
|  |  |  | |  | |  |  |  |  |  |  |  |  |  |  |
| **Education level** |  |  | |  | |  |  |  |  |  |  |  |  |  |  |
| None/Basic | 947 | 411 (43.4) |  | | | 52 (5.5) |  | 426 (45.0) |  | 891 | 678 (76.1) |  | 41 (4.6) |  | 682 (76.5) |
| Secondary | 2404 | 1093 (45.5) |  | | | 108 (4.5) |  | 1131 (47.0) |  | 2347 | 1789 (76.2) |  | 102 (4.3) |  | 1809 (77.1) |
| Undergraduate | 1847 | 874 (47.3) |  | | | 71 (3.8) |  | 903 (48.9) |  | 644 | 498 (77.3) |  | 16 (2.5) |  | 501 (77.8) |
| Postgraduate | 147 | 81 (55.1) | |  | | 6 (4.1) |  | 85 (57.8) |  | 63 | 51 (81.0) |  | 2 (3.2) |  | 51 (81.0) |
| Missing | 84 | 38 (45.2) | |  | | 4 (4.8) |  | 39 (46.4) |  | 11 | 8 (72.7) |  | 1 (9.1) |  | 8 (72.7) |
| **Total** | 5429 | 2497 (46.0) | |  | | 241 (4.4) |  | 2584 (47.6) |  | 3956 | 3024 (76.4) |  | 162 (4.1) |  | 3051 (77.1) |
|  |  |  | |  | |  |  |  |  |  |  |  |  |  |  |
| **Monthly income** |  |  | |  | |  |  |  |  |  |  |  |  |  |  |
| Low | 3826 | 1761 (46) | |  | | 164 (4.3) |  | 1818 (47.5) |  | 3285 | 2531 (77.0) |  | 136 (4.1) |  | 2553 (77.7) |
| Middle | 1241 | 563 (45.4) | | |  | 53 (4.3) |  | 589 (47.5) |  | 582 | 434 (74.6) |  | 22 (3.8) |  | 438 (75.3) |
| High | 69 | 43 (62.3) | |  | | 4 (5.8) |  | 44 (63.8) |  | 46 | 37 (80.4) |  | 3 (6.5) |  | 38 (82.6) |
| Missing | 293 | 130 (44.4) | | |  | 20 (6.8) |  | 133 (45.4) |  | 43 | 22 (51.2) |  | 1 (2.3) |  | 22 (51.2) |
| **Total** | 5429 | 2497 (46.0) | |  | | 241 (4.4) |  | 2584 (47.6) |  | 3956 | 3024 (76.4) |  | 162 (4.1) |  | 3051 (77.1) |
|  |  |  | |  | |  |  |  |  |  |  |  |  |  |  |
| **Employment type** |  |  | |  | |  |  |  |  |  |  |  |  |  |  |
| Formal | 1247 | 584 (46.8) |  | | | 48 (3.8) |  | 605 (48.5) |  | 536 | 418 (78.0) |  | 18 (3.4) |  | 419 (78.2) |
| Informal | 2338 | 1058 (45.3) |  | | | 110 (4.7) |  | 1101 (47.1) |  | 2148 | 1603 (74.6) |  | 103 (4.8) |  | 1617 (75.3) |
| Student | 1096 | 516 (47.1) |  | | | 44 (4.0) |  | 530 (48.4) |  | 906 | 716 (79.0) |  | 22 (2.4) |  | 724 (79.9) |
| Unemployed/Retired | 620 | 281 (45.3) |  | | | 33 (5.3) |  | 289 (46.6) |  | 347 | 273 (78.7) |  | 19 (5.5) |  | 277 (79.8) |
| Missing | 128 | 58 (45.3) | |  | | 6 (4.7) |  | 59 (46.1) |  | 19 | 14 (73.7) |  | 0 (0.0) |  | 14 (73.7) |
| **Total** | 5429 | 2497 (46.0) | |  | | 241 (4.4) |  | 2584 (47.6) |  | 3956 | 3024 (76.4) |  | 162 (4.1) |  | 3051 (77.1) |
|  |  |  | |  | |  |  |  |  |  |  |  |  |  |  |

^a^ June 2021 – November 2021(Delta-dominant period); ^b^ December 2021 – June 2022(Omicron-dominant period). Percentages within each period use participants with available serology results in each category as the denominator; category totals reflect the analytic subset with non-missing data and may differ from other tables.

**Supplementary Table 2c**: Background Characteristics by Seropositivity in Nigeria (JUNE 2021 – MAY 2022)

|  | **(Delta-dominant period)^a^** | | | | | | | | | | | |  | | | **(Omicron-dominant period)^b^** | | | | | | | | | | | | | | | | | | | | | | | |  |  |  |
| --- | --- | --- | --- | --- | --- | --- | --- | --- | --- | --- | --- | --- | --- | --- | --- | --- | --- | --- | --- | --- | --- | --- | --- | --- | --- | --- | --- | --- | --- | --- | --- | --- | --- | --- | --- | --- | --- | --- | --- | --- | --- | --- |
|  |  | **IgG** |  | | **IgM** | | |  | | | **Overall** | | |  | | |  | | | | **IgG** | | | |  | | | | **IgM** | | | |  | | | | **Overall** | | | |  |  |
|  | **Total** | **Positivity** |  | | **Positivity** | | |  | | | **Positivity** | | |  | | | **Total** | | | | **Positivity** | | | |  | | | | **Positivity** | | | |  | | | | **Positivity** | | | |  |  |
|  | **N** | **n (%)** |  | | **n (%)** | | |  | | | **n (%)** | | |  | | | **N** | | | | **n (%)** | | | |  | | | | **n (%)** | | | |  | | | | **n (%)** | | | |  |  |
|  |  |  |  | |  | | |  | | |  | | |  | | |  | | | |  | | | |  | | | |  | | | |  | | | |  | | | |  |  |
| **Recruitment Sites** |  |  |  | |  | | |  | | |  | | |  | | |  | | | |  | | | |  | | | |  | | | |  | | | |  | | | |  |  |
| Shopping Malls | 365 | 243 (66.6) |  | 15 (4.1) | | |  | | | 247 (67.7) | | |  | | | | 283 | | | | 154 (54.4) | | | |  | | | | 10 (3.5) | | | |  | | | | 162 (57.2) | | | |  |  |
| Markets | 1550 | 612 (39.5) |  | 83 (5.4) | | |  | | | 660 (42.6) | | |  | | | | 881 | | | | 617 (70.0) | | | |  | | | | 10 (1.1) | | | |  | | | | 621 (70.5) | | | |  |  |
| Institutions | 653 | 332 (50.8) |  | 48 (7.4) | | |  | | | 356 (54.5) | | |  | | | | 12 | | | | 6 (50.0) | | | |  | | | | 1 (8.3) | | | |  | | | | 6 (50.0) | | | |  |  |
| Community | 1397 | 667 (47.7) |  | 53 (3.8) | | |  | | | 673 (48.2) | | |  | | | | 1036 | | | | 501 (48.4) | | | |  | | | | 17 (1.6) | | | |  | | | | 511 (49.3) | | | |  |  |
| Religious settings |  |  |  | |  | | |  | | |  | | |  | | | 207 | | | | 128 (61.8) | | | |  | | | | 0 (0.0) | | | |  | | | | 128 (61.8) | | | |  |  |
| Health Centre/Hospital | 1037 | 413 (39.8) |  | 43 (4.1) | | |  | | | 425 (41) | | |  | | |  | | | |  | | | |  | | | |  | | | |  | | | |  | | | |  |  |  |
| **Total** | 5002 | 2267(45.3) |  | 242 (4.8) | | |  | | | 2361 (47.2) | | |  | | | | 2419 | | | | 1406 (58.1) | | | |  | | | | 38 (1.6) | | | |  | | | | 1428 (59) | | | |  |  |
|  |  |  |  | |  | | |  | | |  | | |  | | |  | | | |  | | | |  | | | |  | | | |  | | | |  | | | |  |  |
| **Age group (in years)** |  |  |  | |  | | |  | | |  | | |  | | |  | | | |  | | | |  | | | |  | | | |  | | | |  | | | |  |  |
| 4-20 | 719 | 323 (44.9) |  | 36 (5.0) | | |  | | | 338 (47) | | |  | | | 295 | | | | 166 (56.3) | | | |  | | | | 5 (1.7) | | | |  | | | | 167 (56.6) | | | |  |  |  |
| 21-40 | 2695 | 1192 (44.2) |  | 108 (4.0) | | |  | | | 1240 (46) | | |  | | | 1438 | | | | 754 (52.4) | | | |  | | | | 21 (1.5) | | | |  | | | | 768 (53.4) | | | |  |  |  |
| 41-60 | 1204 | 569 (47.3) |  | 72 (6.0) | | |  | | | 595 (49.4) | | |  | | | 559 | | | | 394 (70.5) | | | |  | | | | 9 (1.6) | | | |  | | | | 399 (71.4) | | | |  |  |  |
| 60+ | 383 | 182 (47.5) |  | 26 (6.8) | | |  | | | 187 (48.8) | | |  | | | 119 | | | | 91 (76.5) | | | |  | | | | 2 (1.7) | | | |  | | | | 92 (77.3) | | | |  |  |  |
| Missing | 1 | 1 (100) |  | | 0 (0.0) | | |  | | | 1 (100) | | |  | | | 8 | | | | 1 (12.5) | | | |  | | | | 1 (12.5) | | | |  | | | | 2 (25.0) | | | |  |  |
| **Total** | 5002 | 2267 (45.3) |  | 242 (4.8) | | |  | | | 2361 (47.2) | | |  | | | 2419 | | | | 1406 (58.1) | | | |  | | | | 38 (1.6) | | | |  | | | | 1428 (59.0) | | | |  |  |  |
|  |  |  |  | |  | | |  | | |  | | |  | | |  | | | |  | | | |  | | | |  | | | |  | | | |  | | | |  |  |
| **Gender** |  |  |  | |  | | |  | | |  | | |  | | |  | | | |  | | | |  | | | |  | | | |  | | | |  | | | |  |  |
| Male | 1966 | 953 (48.5) |  | | | 120 (6.1) | | |  | | | 1021 (51.9) | | | | | | 827 | | | | 528 (63.8) | | | |  | | | | 22 (2.7) | | | |  | | | | 540 (65.3) | | | |  |
| Female | 3036 | 1314 (43.3) |  | | | 122 (4.0) | | |  | | | 1340 (44.1) | | | | | | 1592 | | | | 878 (55.2) | | | |  | | | | 16 (1.0) | | | |  | | | | 888 (55.8) | | | |  |
| **Total** | 5002 | 2267 (45.3) |  | | | 242 (4.8) | | |  | | | 2361 (47.2) | | |  | | | | 2419 | | | | 1406 (58.1) | | | |  | | | | 38 (1.6) | | | |  | | | | 1428 (59.0) | | | |
| **Highest educational level** |  |  |  | |  | | |  | | |  | | |  | | |  | | | |  | | | |  | | | |  | | | |  | | | |  | | | |  |  |
| None/Basic | 855 | 354 (41.4) |  | 44 (5.1) | | |  | | | 363 (42.5) | | |  | | | | 584 | | | | 334 (57.2) | | | |  | | | | 8 (1.4) | | | |  | | | | 336 (57.5) | | | |  |  |
| Secondary | 2500 | 1091 (43.6) |  | 117 (4.7) | | |  | | | 1141 (45.6) | | |  | | | | 1340 | | | | 816 (60.9) | | | |  | | | | 18 (1.3) | | | |  | | | | 827 (61.7) | | | |  |  |
| Undergraduate | 1498 | 732 (48.9) |  | 71 (4.7) | | |  | | | 767 (51.2) | | |  | | | | 450 | | | | 233 (51.8) | | | |  | | | | 10 (2.2) | | | |  | | | | 240 (53.3) | | | |  |  |
| Postgraduate | 148 | 89 (60.1) |  | | 9 (6.1) | | |  | | | 89 (60.1) | | |  | | | 39 | | | | 18 (46.2) | | | |  | | | | 2 (5.1) | | | |  | | | | 20 (51.3) | | | |  |  |
| Missing | 1 | 1 (100) |  | | 1 (100) | | |  | | | 1 (100) | | |  | | | 6 | | | | 5 (83.3) | | | |  | | | | 0 (0.0) | | | |  | | | | 5 (83.3) | | | |  |  |
| **Total** | 5002 | 2267 (45.3) |  | | | 242 (4.8) | | |  | | | 2361 (47.2) | | |  | | 2419 | | | | 1406 (58.1) | | | |  | | | | 38 (1.6) | | | |  | | | | 1428 (59.0) | | | |  |  |
|  |  |  |  | |  | | |  | | |  | | |  | | |  | | | |  | | | |  | | | |  | | | |  | | | |  | | | |  |  |
| **Monthly income** |  |  |  | |  | | |  | | |  | | |  | | |  | | | |  | | | |  | | | |  | | | |  | | | |  | | | |  |  |
| Low | 3584 | 1567 (43.7) |  | 164 (4.6) | | |  | | | 1620 (45.2) | | |  | | | 1667 | | | | 945 (56.7) | | | |  | | | | 22 (1.3) | | | |  | | | | 959 (57.5) | | | |  |  |  |
| Middle | 208 | 120 (57.7) |  | 26 (12.5) | | |  | | | 143 (68.8) | | |  | | | 682 | | | | 420 (61.6) | | | |  | | | | 13 (1.9) | | | |  | | | | 425 (62.3) | | | |  |  |  |
| High | 1209 | 580 (48.0) |  | | 52 (4.3) | | |  | | | 598 (49.5) | | |  | | 66 | | | | 37 (56.1) | | | |  | | | | 3 (4.5) | | | |  | | | | 40 (60.6) | | | |  |  |  |
| Missing | 1 | 0 (0.0) |  | | 0 (0.0) | | |  | | | 0 (0.0) | | |  | | | 4 | | | | 4 (100) | | | |  | | | | 0 (0.0) | | | |  | | | | 4 (100) | | | |  |  |
| **Total** | 5002 | 2267 (45.3) |  | 242 (4.8) | | |  | | | 2361 (47.2) | | |  | | | 2419 | | | | 1406 (58.1) | | | |  | | | | 38 (1.6) | | | |  | | | | 1428 (59.0) | | | |  |  |  |
|  |  |  |  | |  | | |  | | |  | | |  | | |  | | | |  | | | |  | | | |  | | | |  | | | |  | | | |  |  |
| **Employment type** |  |  |  | |  | | |  | | |  | | |  | | |  | | | |  | | | |  | | | |  | | | |  | | | |  | | | |  |  |
| Formal | 1032 | 527 (51.1) |  | 51 (4.9) | | |  | | | 538 (52.1) | | |  | | | 331 | | | | 170 (51.4) | | | |  | | | | 9 (2.7) | | | |  | | | | 177 (53.5) | | | |  |  |  |
| Informal | 2694 | 1128 (41.9) |  | 127 (4.7) | | |  | | | 1182 (43.9) | | |  | | | 1509 | | | | 945 (62.6) | | | |  | | | | 20 (1.3) | | | |  | | | | 954 (63.2) | | | |  |  |  |
| Student | 809 | 349 (43.1) |  | 31 (3.8) | | |  | | | 358 (44.3) | | |  | | | 218 | | | | 108 (49.5) | | | |  | | | | 2 (0.9) | | | |  | | | | 110 (50.5) | | | |  |  |  |
| Unemployed/Retired | 467 | 263 (56.3) |  | 33 (7.1) | | |  | | | 283 (60.6) | | |  | | | 352 | | | | 176 (50.0) | | | |  | | | | 7 (2.0) | | | |  | | | | 180 (51.1) | | | |  |  |  |
| Missing |  |  |  | |  | | |  | | |  | | |  | | | 9 | | | | 7 (77.8) | | | |  | | | | 0 (0.0) | | | |  | | | | 7 (77.8) | | | |  |  |
| **Total** | 5002 | 2267 (45.3) | | 242 (4.8) | | |  | | | 2361 (47.2) | | | | | | 2419 | | | | 1406 (58.1) | | | | | | | | 38 (1.6) | | | |  | | | | 1428 (59.0) | | | |  |  |  |
|  |  |  |  | |  | | |  | | |  | | |  | | |  | | | |  | | | |  | | | |  | | | |  | | | |  | | | |  |  |

^a^ June 2021 – November 2021 (Delta-dominant period); ^b^ December 2021 – June 2022 (Omicron-dominant period). Percentages within each period use participants with available serology results in each category as the denominator; category totals reflect the analytic subset with non-missing data and may differ from other tables.

**Supplementary Table 3a: Log-binomial regression analysis of factors associated with IgG positivity by country (Delta-dominant period)**

|  | **Burkina Faso** | |  | **Ghana** | |  | **Nigeria** | |
| --- | --- | --- | --- | --- | --- | --- | --- | --- |
|  | **PR (95% CI)** | **P-value** |  | **PR (95% CI)** | **P-value** |  | **PR (95% CI)** | **P-value** |
| **Recruitment site** |  |  |  |  |  |  |  |  |
| Institutions | **1 (ref)** |  |  | **1 (ref)** |  |  | **1 (ref)** |  |
| Shopping Malls |  |  |  | 1.2 (1.0 - 1.3) | 0.005 |  | 1.3 (1.2 - 1.5) | <0.001 |
| Markets/Motor parks | 0.6 (0.5 - 0.7) | <0.001 |  | 1.1 (1.0 - 1.2) | 0.010 |  | 0.8 (0.7 - 0.9) | <0.001 |
| Community | - |  |  | 0.9 (0.8 - 1.0) | 0.238 |  | 0.9 (0.9 - 1.0) | 0.187 |
| Religious setting | 1.3 (1.0 - 1.6) | 0.042 |  | 1.2 (1.1 - 1.3) | <0.001 |  | - |  |
| Health centers/Hospital | 0.5 (0.1 - 3.1) | 0.463 |  | 0.8 (0.8 - 1.0) | 0.006 |  | 0.8 (0.7 - 0.9) | <0.001 |
| **Age group (in years)** |  |  |  |  |  |  |  |  |
| 4-20 | **1 (ref)** |  |  | **1 (ref)** |  |  | **1 (ref)** |  |
| 21-40 | 1.2 (0.9 - 1.6) | 0.149 |  | 1.1 (1.0 - 1.2) | 0.297 |  | 1.0 (0.9 - 1.1) | 0.739 |
| 41-60 | 1.4 (1.0 - 1.9) | 0.026 |  | 1.2 (1.1 - 1.3) | 0.001 |  | 1.1 (1.0 - 1.2) | 0.323 |
| 60+ | 1.7 (1.2 - 2.5) | 0.002 |  | 1.3 (1.1 - 1.5) | <0.001 |  | 1.1 (0.9 - 1.2) | 0.407 |
| **Gender** |  |  |  |  |  |  |  |  |
| Male | **1 (ref)** |  |  | **1 (ref)** |  |  | **1 (ref)** |  |
| Female | 0.8 (0.7 - 1.0) | 0.010 |  | 1.1 (1.1 - 1.2) | <0.001 |  | 0.9 (0.8 - 0.9) | <0.001 |
|  |  |  |  |  |  |  |  |  |
| **Education level** |  |  |  |  |  |  |  |  |
| None/Basic | **1 (ref)** |  |  | **1 (ref)** |  |  | **1 (ref)** |  |
| Secondary | 1.0 (0.9 - 1.2) | 0.688 |  | 1.0 (1.0 - 1.1) | 0.283 |  | 1.1 (1.0 - 1.2) | 0.259 |
| Undergraduate | 1.4 (1.1 - 1.7) | 0.007 |  | 1.1 (1.0 - 1.2) | 0.052 |  | 1.2 (1.1 - 1.3) | 0.001 |
| Postgraduate | 1.4 (1.1 - 1.9) | 0.014 |  | 1.3 (1.1 - 1.5) | 0.004 |  | 1.5 (1.2 - 1.7) | <0.001 |
| **Monthly income** |  |  |  |  |  |  |  |  |
| Low | **1 (ref)** |  |  | **1 (ref)** |  |  | **1 (ref)** |  |
| Middle | 1.1 (0.9 - 1.3) | 0.328 |  | 1.0 (0.9 - 1.1) | 0.686 |  | 1.3 (1.2 - 1.5) | <0.001 |
| High | 1.4 (1.1 - 1.7) | 0.004 |  | 1.4 (1.1 - 1.6) | 0.001 |  | 1.1 (1.0 - 1.2) | 0.009 |
| **Employment type** |  |  |  |  |  |  |  |  |
| Formal | **1 (ref)** |  |  | **1 (ref)** |  |  | **1 (ref)** |  |
| Informal | 0.8 (0.6 - 1.0) | 0.017 |  | 1.0 (0.9 - 1.0) | 0.364 |  | 0.8 (0.8 - 0.9) | <0.001 |
| Student | 0.8 (0.6 - 1.0) | 0.036 |  | 1.0 (0.9 - 1.1) | 0.904 |  | 0.8 (0.8 - 0.9) | 0.001 |
| Unemployed/Retired | 0.6 (0.4 - 1.0) | 0.054 |  | 1.0 (0.9 - 1.1) | 0.540 |  | 1.1 (1.0 - 1.2) | 0.054 |

PR = prevalence ratio (log-binomial regression); ref = reference category; CI = confidence interval.

**Supplementary table 3b: Log-binomial regression analysis of factors associated with seropositivity (Omicron-dominant period)**

|  | **Burkina Faso** | |  | **Ghana** | |  | **Nigeria** | |
| --- | --- | --- | --- | --- | --- | --- | --- | --- |
|  | **PR (95% CI)** | **P-value** |  | **PR (95% CI)** | **P-value** |  | **PR (95% CI)** | **P-value** |
| **Recruitment site** |  |  |  |  |  |  |  |  |
| Institutions |  |  |  | **1 (ref)** |  |  | **1 (ref)** |  |
| Shopping Malls |  |  |  |  |  |  | 1.1 (0.6 - 1.9) | 0.773 |
| Markets/Lorry parks |  |  |  | 0.9 (0.9 - 1.0) | <0.001 |  | 1.4 (0.8 - 2.5) | 0.245 |
| Community |  |  |  | 0.9 (0.9 - 1.0) | <0.001 |  | 1.0 (0.5 - 1.7) | 0.909 |
| Religious settings |  |  |  | 0.9 (0.8 - 1.0) | 0.014 |  | 1.2 (0.7 - 2.2) | 0.470 |
| Health centers/Hospitals |  |  |  | 0.7 (0.6 - 0.9) | 0.004 |  |  |  |
| **Age group (in years)** |  |  |  |  |  |  |  |  |
| 4-20 | **1 (ref)** |  |  | **1 (ref)** |  |  | **1 (ref)** |  |
| 21-40 | 1.2 (0.8 - 1.9) | 0.411 |  | 1.0 (1.0 - 1.1) | 0.369 |  | 0.9 (0.8 - 1.0) | 0.217 |
| 41-60 | 1.6 (1.0 - 2.5) | 0.060 |  | 1.1 (1.0 - 1.2) | 0.002 |  | 1.3 (1.1 - 1.4) | <0.001 |
| 60+ | 1.7 (1.0 - 2.7) | 0.052 |  | 1.2 (1.1 - 1.2) | <0.001 |  | 1.4 (1.2 - 1.6) | <0.001 |
| **Gender** |  |  |  |  |  |  |  |  |
| Male | **1 (ref)** |  |  | **1 (ref)** |  |  | **1 (ref)** |  |
| Female | 1.1 (0.9 - 1.3) | 0.476 |  | 1.1 (1.1 - 1.2) | <0.001 |  | 0.86 (0.8 - 0.92) | <0.001 |
|  |  |  |  |  |  |  |  |  |
| **Educational level** |  |  |  |  |  |  |  |  |
| None/Basic | **1 (ref)** |  |  | **1 (ref)** |  |  | **1 (ref)** |  |
| Secondary | 0.9 (0.7 - 1.1) | 0.335 |  | 1.0 (0.96 - 1.04) | 0.938 |  | 1.1 (1.0 - 1.2) | 0.135 |
| Undergraduate | 0.6 (0.4 - 1.2) | 0.159 |  | 1.1 (0.9 - 1.4) | 0.571 |  | 0.9 (0.8 - 1.0) | 0.086 |
| Postgraduate | 0.5 (0.2 - 1.1) | 0.077 |  | 1.1 (0.9 - 1.2) | 0.333 |  | 0.8 (0.6 - 1.1) | 0.225 |
| **Monthly Income** |  |  |  |  |  |  |  |  |
| Low | **1 (ref)** |  |  | **1 (ref)** |  |  | **1 (ref)** |  |
| Middle | 1.1 (0.9 - 1.4) | 0.198 |  | 0.97 (0.9 - 1.01) | 0.209 |  | 1.1 (1.0 - 1.2) | 0.025 |
| High | 1.4 (1.1 - 1.8) | 0.007 |  | 1.0 (0.9 - 1.2) | 0.557 |  | 1.0 (0.8 - 1.2) | 0.920 |
| **Employment type** |  |  |  |  |  |  |  |  |
| Formal | **1 (ref)** |  |  | **1 (ref)** |  |  | **1 (ref)** |  |
| Informal | 0.8 (0.6 - 1.1) | 0.193 |  | 0.96 (0.9 - 1.0) | 0.093 |  | 1.2 (1.1 - 1.4) | 0.001 |
| Student | 0.4 (0.2 - 0.8) | 0.006 |  | 1.0 (0.95 - 1.1) | 0.642 |  | 1.0 (0.8 - 1.1) | 0.678 |
| Unemployed/Retired | 0.8 (0.5 - 1.3) | 0.442 |  | 1.0 (0.9 - 1.1) | 0.808 |  | 1.0 (0.8 - 1.1) | 0.722 |

PR = prevalence ratio (log-binomial regression); ref = reference category; CI = confidence interval.

**Supplementary Table 4**: Clinical symptoms in the last 4 weeks and associated seropositivity by country from June 2021- June 2022

|  | **BURKINA FASO** | | | | | | | |  | | | **GHANA** | | | | | | | | | | |  | | | **NIGERIA** | | | | | | | | | | | | | | | |  |  |
| --- | --- | --- | --- | --- | --- | --- | --- | --- | --- | --- | --- | --- | --- | --- | --- | --- | --- | --- | --- | --- | --- | --- | --- | --- | --- | --- | --- | --- | --- | --- | --- | --- | --- | --- | --- | --- | --- | --- | --- | --- | --- | --- | --- |
|  | **Overall (IgG/IgM)** | | | | | | | |  | | | **Overall (IgG/IgM)** | | | | | | | | | | |  | | | **Overall (IgG/IgM)** | | | | | | | | | | | | | | | |  |  |
|  | **Positive** | |  | **Negative** |  | | **Total** | **P value** | | |  | | | **Positive** | |  | | **Negative** |  | **Total** | | **P value** | | |  | | | **Positive** | |  | | **Negative** | |  | | | **Total** | | | **P value** | | |  |
|  | **n (%)** | |  | **n (%)** |  | | **n (%)** |  | | |  | | | **n (%)** | |  | | **n (%)** |  | **n (%)** | |  | | |  | | | **n (%)** | |  | | | **n (%)** | |  | | | **n (%)** | | |  | | |
| **Symptoms** | | |  |  |  | |  |  | | |  | | |  | |  | |  |  |  | |  | | |  | | |  | |  | | |  | |  | | |  | | |  | | |
| **Fever** |  | |  |  |  | |  |  | | |  | | |  | |  | |  |  |  | |  | | |  | | |  | |  | | |  | |  | | |  | | |  | | |
| Yes | 223 (29.9) | | | 492 (28.8) | | 715 (29.1) | | 0.587 | |  | | | 512 (19.9) | | | | 472 (17.8) | | 984 (18.8) | | 0.061 | | |  | | | 608 (16.0) | | | | 539 (14.8) | | | | | 1147 (15.5) | | | 0.15 | | |  |  |
| No | 524 (70.1) | | | 1218 (71.2) | | 1742 (70.9) | | | |  | | | 2067 (80.1) | | | | 2176 (82.2) | | 4243 (81.2) | | | | |  | | | 3181 (84.0) | | | | 3093 (85.2) | | | | | 6274 (84.5) | | | | | |  |  |
| **Total** | 747 (100) | |  | 1710 (100) | | 2457 (100) | | | |  | | | 2579 (100) | | | | 2648 (100) | | 5227 (100) | | | | |  | | | 3789 (100) | | | | 3632 (100) | | | | | 7421 (100) | | | | | |  |  |
|  |  | |  |  |  | |  |  | | |  | | |  | |  | |  |  |  | |  | | |  | | |  | |  | | |  | |  | | |  | | |  | | |
| **Shortness of breath** | | |  |  |  | |  |  | | |  | | |  | |  | |  |  |  | |  | | |  | | |  | |  | | |  | |  | | |  | | |  | | |
| Yes | 66 (8.8) | |  | 141 (8.2) |  | | 207 (8.4) | 0.628 | | |  | | | 95 (3.7) | |  | | 88 (3.3) |  | 183 (3.5) | | 0.479 | | |  | | | 88 (2.3) | |  | | | 43 (1.2) | |  | | | 131 (1.8) | | | <0.001 | | |
| No | 681 (91.2) | | | 1569 (91.8) | | 2250 (91.6) | | | |  | | | 2484 (96.3) | | | | 2560 (96.7) | | 5044 (96.5) | | | | |  | | | 3701 (97.7) | | | | 3589 (98.8) | | | | | 7290 (98.2) | | | | | |  |  |
| **Total** | 747 (100) | |  | 1710 (100) | | 2457 (100) | | | |  | | | 2579 (100) | | | | 2648 (100) | | 5227 (100) | | | | |  | | | 3789 (100) | | | | 3632 (100) | | | | | 7421 (100) | | | | | |  |  |
|  |  | |  |  |  | |  |  | | |  | | |  | |  | |  |  |  | |  | | |  | | |  | |  | | |  | |  | | |  | | |  | | |
| **Cough** |  | |  |  |  | |  |  | | |  | | |  | |  | |  |  |  | |  | | |  | | |  | |  | | |  | |  | | |  | | |  | | |
| Yes | 101 (13.5) | | | 201 (11.8) | | 302 (12.3) | | 0.22 | |  | | | 459 (17.8) | | | | 480 (18.1) | | 939 (18.0) | | 0.757 | | |  | | | 404 (10.7) | | | | 211 (5.8) | | |  | | | 615 (8.3) | | | <0.001 | | |  |
| No | 646 (86.5) | | | 1509 (88.2) | | 2155 (87.7) | | | |  | | | 2120 (82.2) | | | | 2168 (81.9) | | 4288 (82.0) | | | | |  | | | 3385 (89.3) | | | | 3421 (94.2) | | | | | 6806 (91.7) | | | | | |  |  |
| **Total** | 747 (100) | |  | 1710 (100) | | 2457 (100) | | | |  | | | 2579 (100) | | | | 2648 (100) | | 5227 (100) | | | | |  | | | 3789 (100) | | | | 3632 (100) | | | | | 7421 (100) | | | | | |  |  |
|  |  | |  |  |  | |  |  | | |  | | |  | |  | |  |  |  | |  | | |  | | |  | |  | | |  | |  | | |  | | |  | | |
| **Loss of smell/taste** | | |  |  |  | |  |  | | |  | | |  | |  | |  |  |  | |  | | |  | | |  | |  | | |  | |  | | |  | | |  | | |
| Yes | 28 (3.7) | |  | 45 (2.6) |  | | 73 (3.0) | 0.134 | | |  | | | 132 (5.1) | |  | | 112 (4.2) |  | 244 (4.7) | | 0.128 | | |  | | | 105 (2.8) | |  | | | 44 (1.2) | |  | | | 149 (2.0) | | | <0.001 | | |
| No | 719 (96.3) | | | 1665 (97.4) | | 2384 (97.0) | | | |  | | | 2447 (94.9) | | | | 2536 (95.8) | | 4983 (95.3) | | | | |  | | | 3684 (97.2) | | | | 3588 (98.8) | | | | | 7272 (98.0) | | | | | |  |  |
| **Total** | 747 (100) | |  | 1710 (100) | | 2457 (100) | | | |  | | | 2579 (100) | | | | 2648 (100) | | 5227 (100) | | | | |  | | | 3789 (100) | | | | 3632 (100) | | | | | 7421 (100) | | | | | |  |  |
|  |  | |  |  |  | |  |  | | |  | | |  | |  | |  |  |  | |  | | |  | | |  | |  | | |  | |  | | |  | | |  | | |
| **Headache** | | |  |  |  | |  |  | | |  | | |  | |  | |  |  |  | |  | | |  | | |  | |  | | |  | |  | | |  | | |  | | |
| Yes | 118 (15.8) | | | 236 (13.8) | | 354 (14.4) | | 0.195 | |  | | | 1027 (39.8) | | | | 1072 (40.5) | | 2099 (40.2) | | 0.626 | | |  | | | 720 (19.0) | | | | 539 (14.8) | | | | | 1259 (17.0) | | | <0.001 | | |  |  |
| No | 629 (84.2) | | | 1474 (86.2) | | 2103 (85.6) | | | |  | | | 1552 (60.2) | | | | 1576 (59.5) | | 3128 (59.8) | | | | |  | | | 3069 (81.0) | | | | 3093 (85.2) | | | | | 6162 (83.0) | | | | | |  |  |
| **Total** | 747 (100) | |  | 1710 (100) | | 2457 (100) | | | |  | | | 2579 (100) | | | | 2648 (100) | | 5227 (100) | | | | |  | | | 3789 (100) | | | | 3632 (100) | | | | | 7421 (100) | | | | | |  |  |
|  |  | |  |  |  | |  |  | | |  | | |  | |  | |  |  |  | |  | | |  | | |  | |  | | |  | |  | | |  | | |  | | |
| **Diarrhea** |  | |  |  |  | |  |  | | |  | | |  | |  | |  |  |  | |  | | |  | | |  | |  | | |  | |  | | |  | | |  | | |
| Yes | 11 (1.5) | |  | 41 (2.4) |  | | 52 (2.1) | 0.143 | | |  | | | 118 (4.6) | |  | | 114 (4.3) |  | 232 (4.4) | | 0.635 | | |  | | | 63 (1.7) | |  | | | 43 (1.2) | |  | | | 106 (1.4) | | | 0.08 | | |
| No | 736 (98.5) | | | 1669 (97.6) | | 2405 (97.9) | | | |  | | | 2461 (95.4) | | | | 2534 (95.7) | | 4995 (95.6) | | | | |  | | | 3726 (98.3) | | | | 3589 (98.8) | | | | | 7315 (98.6) | | | | | |  |  |
| **Total** | 747 (100) | |  | 1710 (100) | | 2457 (100) | | | |  | | | 2579 (100) | | | | 2648 (100) | | 5227 (100) | | | | |  | | | 3789 (100) | | | | 3632 (100) | | | | | 7421 (100) | | | | | |  |  |
|  |  | |  |  |  | |  |  | | |  | | |  | |  | |  |  |  | |  | | |  | | |  | |  | | |  | |  | | |  | | |  | | |
| **Sore throat** | | |  |  |  | |  |  | | |  | | |  | |  | |  |  |  | |  | | |  | | |  | |  | | |  | |  | | |  | | |  | | |
| Yes | 9 (1.2) | |  | 14 (0.8) |  | | 23 (0.9) | 0.361 | | |  | | | 162 (6.3) | |  | | 171 (6.5) |  | 333 (6.4) | | 0.794 | | |  | | | 116 (3.1) | |  | | | 43 (1.2) | |  | | | 159 (2.1) | | | <0.001 | | |
| No | 738 (98.8) | | | 1696 (99.2) | | 2434 (99.1) | | | |  | | | 2417 (93.7) | | | | 2477 (93.5) | | 4894 (93.6) | | | | |  | | | 3673 (96.9) | | | | 3589 (98.8) | | | | | 7262 (97.9) | | | | | |  |  |
| **Total** | 747 (100) | |  | 1710 (100) | | 2457 (100) | | | |  | | | 2579 (100) | | | | 2648 (100) | | 5227 (100) | | | | |  | | | 3789 (100) | | | | 3632 (100) | | | | | 7421 (100) | | | | | |  |  |
|  |  | |  |  |  | |  |  | | |  | | |  | |  | |  |  |  | |  | | |  | | |  | |  | | |  | |  | | |  | | |  | | |
| **Vomiting** | |  |  |  |  | |  |  | | |  | | |  | |  | |  |  |  | |  | | |  | | |  | |  | | |  | |  | | |  | | |  | | |
| Yes | 16 (2.1) | |  | 61 (3.6) |  | | 77 (3.1) | 0.062 | | |  | | | 69 (2.7) | |  | | 72 (2.7) |  | 141 (2.7) | | 0.923 | | |  | | | 61 (1.6) | |  | | | 32 (0.9) | |  | | | 93 (1.3) | | | 0.01 | | |
| No | 731 (97.9) | | | 1649 (96.4) | | 2380 (96.9) | | | |  | | | 2510 (97.3) | | | | 2576 (97.3) | | 5086 (97.3) | | | | |  | | | 3728 (98.4) | | | | 3600 (99.1) | | | | | 7328 (98.7) | | | | | |  |  |
| **Total** | 747 (100) | |  | 1710 (100) | | 2457 (100) | | | |  | | | 2579 (100) | | | | 2648 (100) | | 5227 (100) | | | | |  | | | 3789 (100) | | | | 3632 (100) | | | | | 7421 (100) | | | | | |  |  |
|  |  | |  |  |  | |  |  | | |  | | |  | |  | |  |  |  | |  | | |  | | |  | |  | | |  | |  | | |  | | |  | | |
| **Runny nose** | | |  |  |  | |  |  | | |  | | |  | |  | |  |  |  | |  | | |  | | |  | |  | | |  | |  | | |  | | |  | | |
| Yes | 109 (14.6) | | | 217 (12.7) | | 326 (13.3) | | 0.201 | |  | | | 314 (12.2) | | | | 332 (12.5) | | 646 (12.4) | | 0.691 | | |  | | | 253 (6.7) | |  | | 123 (3.4) | | |  | | | 376 (5.1) | | | <0.001 | | |  |
| No | 638 (85.4) | | | 1493 (87.3) | | 2131 (86.7) | | | |  | | | 2265 (87.8) | | | | 2316 (87.5) | | 4581 (87.6) | | | | |  | | | 3536 (93.3) | | | | 3509 (96.6) | | | | | 7045 (94.9) | | | | | |  |  |
| **Total** | 747 (100) | |  | 1710 (100) | | 2457 (100) | | | |  | | | 2579 (100) | | | | 2648 (100) | | 5227 (100) | | | | |  | | | 3789 (100) | | | | 3632 (100) | | | | | 7421 (100) | | | | | |  |  |
|  |  | |  |  |  | |  |  | | |  | | |  | |  | |  |  |  | |  | | |  | | |  | |  | | |  | |  | | |  | | |  | | |
| **Skin rash** | |  |  |  |  | |  |  | | |  | | |  | |  | |  |  |  | |  | | |  | | |  | |  | | |  | |  | | |  | | |  | | |
| Yes | 11 (1.5) | |  | 33 (1.9) |  | | 44 (1.8) | 0.432 | | |  | | | 71 (2.8) | |  | | 73 (2.8) |  | 144 (2.8) | | 0.993 | | |  | | | 40 (1.1) | |  | | | 27 (0.7) | |  | | | 67 (0.9) | | | 0.16 | | |
| No | 736 (98.5) | | | 1677 (98.1) | | 2413 (98.2) | | | |  | | | 2508 (97.2) | | | | 2575 (97.2) | | 5083 (97.2) | | | | |  | | | 3749 (98.9) | | | | 3605 (99.3) | | | | | 7354 (99.1) | | | | | |  |  |
| **Total** | 747 (100) | |  | 1710 (100) | | 2457 (100) | | | |  | | | 2579 (100) | | | | 2648 (100) | | 5227 (100) | | | | |  | | | 3789 (100) | | | | 3632 (100) | | | | | 7421 (100) | | | | | |  |  |
|  |  | |  |  |  | |  |  | | |  | | |  | |  | |  |  |  | |  | | |  | | |  | |  | | |  | |  | | |  | | |  | | |
| **Other** |  | |  |  |  | |  |  | | |  | | |  | |  | |  |  |  | |  | | |  | | |  | |  | | |  | |  | | |  | | |  | | |
| Yes | 5 (0.7) | |  | 15 (0.9) |  | | 20 (0.8) | 0.598 | | |  | | | 51 (2.0) | |  | | 40 (1.5) |  | 91 (1.7) | | 0.197 | | |  | | | 40 (1.1) | |  | | | 23 (0.6) | |  | | | 63 (0.8) | | | 0.05 | | |
| No | 742 (99.3) | | | 1695 (99.1) | | 2437 (99.2) | | | |  | | | 2528 (98) | |  | | 2608 (98.5) | | 5136 (98.3) | | | | |  | | | 3749 (98.9) | | | | 3609 (99.4) | | | | | 7358 (99.2) | | | | | |  |  |
| **Total** | 747 (100) | |  | 1710 (100) | | 2457 (100) | | | |  | | | 2579 (100) | | | | 2648 (100) | | 5227 (100) | | | | |  | | | 3789 (100) | | | | 3632 (100) | | | | | 7421 (100) | | | | | |  |  |
|  |  | |  |  |  | |  |  | | |  | | |  | |  | |  |  |  | |  | | |  | | |  | |  | | |  | |  | | |  | | |  | | |

P-values were generated from chi-square and Fisher’s exact tests.

**Supplementary Table 5**: Diagnosed Diseases and their association with seropositivity by country from June 2021- June 2022

|  | | **BURKINA FASO** | | | | | | | | | | | | |  | | | **GHANA** | | | | | | | | | | | | | | | | | |  | | |  | | |  | | | **NIGERIA** | | | | | | | | | | | | |  |
| --- | --- | --- | --- | --- | --- | --- | --- | --- | --- | --- | --- | --- | --- | --- | --- | --- | --- | --- | --- | --- | --- | --- | --- | --- | --- | --- | --- | --- | --- | --- | --- | --- | --- | --- | --- | --- | --- | --- | --- | --- | --- | --- | --- | --- | --- | --- | --- | --- | --- | --- | --- | --- | --- | --- | --- | --- | --- | --- |
|  | |  | |  | **Overall**  **(IgG/IgM)** | | |  | | |  | | |  | |  | | |  | | |  | | | **Overall (IgG/IgM)** | | |  | | |  | | | |  | |  | | |  | | |  | | | **Overall (IgG/IgM)** | | | | |  | |  | | |  | | |
|  | **Positive** | | |  | **Negative** | |  | | | **Total** | | | **P-value** | | | |  | | | **Positive** | | |  | | | **Negative** | | |  | | | **Total** | | **P-value** | | | |  | | | **Positive** | | |  | | | **Negative** |  | | **Total** | | | | **P-value** | | |  |  |
|  | | **n (%)** | |  | **n (%)** |  | | | **n (%)** | | |  | | |  | | | **n (%)** | | |  | | | **n (%)** | | |  | | | **n (%)** | | |  | | |  | | | **n (%)** | | |  | | | **n (%)** | | | |  | | | **n (%)** | | |  | | |  |
| **Diseases** | |  | |  |  |  | | |  | | |  | | |  | | |  | | |  | | |  | | |  | | |  | | |  | | |  | | |  | | |  | | |  | | | |  | | |  | | |  | | |  |
| **Heart Disease** | | |  |  |  |  | | |  | | |  | | |  | | |  | | |  | | |  | | |  | | |  | | |  | | |  | | |  | | |  | | |  | | | |  | | |  | | |  | | |  |
| Yes | | 77 (10.3) | |  | 159 (9.3) |  | | | 236 (9.6) | | | 0.435 | | |  | | | 262 (10.2) | | |  | | | 172 (6.5) | | |  | | | 434 (8.3) | | | <0.001 | | |  | | | 182 (4.8) | | |  | | | 128 (3.5) | | | |  | | | 310 (4.2) | | | 0.006 | | |  |
| No | | 670 (89.7) | |  | 1551 (90.7) |  | | | 2221 (90.4) | | |  | | |  | | | 2317 (89.8) | | |  | | | 2476 (93.5) | | |  | | | 4793 (91.7) | | |  | | |  | | | 3607 (95.2) | | |  | | | 3504 (96.5) | | | |  | | | 7111 (95.8) | | |  | | |  |
| **Total** | | 747 (100) | |  | 1710 (100) |  | | | 2457 (100) | | |  | | |  | | | 2579 (100) | | |  | | | 2648 (100) | | |  | | | 5227 (100) | | |  | | |  | | | 3789 (100) | | |  | | | 3632 (100) | | | |  | | | 7421 (100) | | |  | | |  |
|  | |  | |  |  |  | | |  | | |  | | |  | | |  | | |  | | |  | | |  | | |  | | |  | | |  | | |  | | |  | | |  | | | |  | | |  | | |  | | |  |
| **Obesity** | |  | |  |  |  | | |  | | |  | | |  | | |  | | |  | | |  | | |  | | |  | | |  | | |  | | |  | | |  | | |  | | | |  | | |  | | |  | | |  |
| Yes | | 9 (1.2) | |  | 31 (1.8) |  | | | 40 (1.6) | | | 0.273 | | |  | | | 39 (1.5) | | |  | | | 26 (1.0) | | |  | | | 65 (1.2) | | | 0.084 | | |  | | | 17 (0.4) | | |  | | | 12 (0.3) | | | |  | | | 29 (0.4) | | | 0.414 | | |  |
| No | | 738 (98.8) | |  | 1679 (98.2) |  | | | 2417 (98.4) | | |  | | |  | | | 2540 (98.5) | | |  | | | 2622 (99) | | |  | | | 5162 (98.8) | | |  | | |  | | | 3772 (99.6) | | |  | | | 3620 (99.7) | | | |  | | | 7392 (99.6) | | |  | | |  |
| **Total** | | 747 (100) | |  | 1710 (100) |  | | | 2457 (100) | | |  | | |  | | | 2579 (100) | | |  | | | 2648 (100) | | |  | | | 5227 (100) | | |  | | |  | | | 3789 (100) | | |  | | | 3632 (100) | | | |  | | | 7421 (100) | | |  | | |  |
|  | |  | |  |  |  | | |  | | |  | | |  | | |  | | |  | | |  | | |  | | |  | | |  | | |  | | |  | | |  | | |  | | | |  | | |  | | |  | | |  |
| **Kidney Disease** | | |  |  |  |  | | |  | | |  | | |  | | |  | | |  | | |  | | |  | | |  | | |  | | |  | | |  | | |  | | |  | | | |  | | |  | | |  | | |  |
| Yes | | 3 (0.4) | |  | 2 (0.1) |  | | | 5 (0.2) | | | 0.15 | | |  | | | 10 (0.4) | | |  | | | 7 (0.3) | | |  | | | 17 (0.3) | | | 0.433 | | |  | | | 4 (0.1) | | |  | | | 8 (0.2) | | | |  | | | 12 (0.2) | | | 0.219 | | |  |
| No | | 744 (99.6) | |  | 1708 (99.9) |  | | | 2452 (99.8) | | |  | | |  | | | 2569 (99.6) | | |  | | | 2641 (99.7) | | |  | | | 5210 (99.7) | | |  | | |  | | | 3785 (99.9) | | |  | | | 3624 (99.8) | | | |  | | | 7409 (99.8) | | |  | | |  |
| **Total** | | 747 (100) | |  | 1710 (100) |  | | | 2457 (100) | | |  | | |  | | | 2579 (100) | | |  | | | 2648 (100) | | |  | | | 5227 (100) | | |  | | |  | | | 3789 (100) | | |  | | | 3632 (100) | | | |  | | | 7421 (100) | | |  | | |  |
|  | |  | |  |  |  | | |  | | |  | | |  | | |  | | |  | | |  | | |  | | |  | | |  | | |  | | |  | | |  | | |  | | | |  | | |  | | |  | | |  |
| **Asthma** | |  | |  |  |  | | |  | | |  | | |  | | |  | | |  | | |  | | |  | | |  | | |  | | |  | | |  | | |  | | |  | | | |  | | |  | | |  | | |  |
| Yes | | 18 (2.4) | |  | 45 (2.6) |  | | | 63 (2.6) | | | 0.749 | | |  | | | 52 (2.0) | | |  | | | 57 (2.2) | | |  | | | 109 (2.1) | | | 0.73 | | |  | | | 42 (1.1) | | |  | | | 52 (1.4) | | | |  | | | 94 (1.3) | | | 0.213 | | |  |
| No | | 729 (97.6) | |  | 1665 (97.4) |  | | | 2394 (97.4) | | |  | | |  | | | 2527 (98) | | |  | | | 2591 (97.8) | | |  | | | 5118 (97.9) | | |  | | |  | | | 3747 (98.9) | | |  | | | 3580 (98.6) | | | |  | | | 7327 (98.7) | | |  | | |  |
| **Total** | | 747 (100) | |  | 1710 (100) |  | | | 2457 (100) | | |  | | |  | | | 2579 (100) | | |  | | | 2648 (100) | | |  | | | 5227 (100) | | |  | | |  | | | 3789 (100) | | |  | | | 3632 (100) | | | |  | | | 7421 (100) | | |  | | |  |
|  | |  | |  |  |  | | |  | | |  | | |  | | |  | | |  | | |  | | |  | | |  | | |  | | |  | | |  | | |  | | |  | | | |  | | |  | | |  | | |  |
| **TB** | |  | |  |  |  | | |  | | |  | | |  | | |  | | |  | | |  | | |  | | |  | | |  | | |  | | |  | | |  | | |  | | | |  | | |  | | |  | | |  |
| Yes | | 1 (0.1) | |  | 11 (0.6) |  | | | 12 (0.5) | | | 0.096 | | |  | | | 7 (0.3) | | |  | | | 10 (0.4) | | |  | | | 17 (0.3) | | |  | | |  | | | 4 (0.1) | | |  | | | 2 (0.1) | | | |  | | | 6 (0.1) | | | 0.444 | | |  |
| No | | 746 (99.9) | |  | 1699 (99.4) |  | | | 2445 (99.5) | | |  | | |  | | | 2572 (99.7) | | |  | | | 2638 (99.6) | | |  | | | 5210 (99.7) | | | 0.5 | | |  | | | 3785 (99.9) | | |  | | | 3630 (99.9) | | | |  | | | 7415 (99.9) | | |  | | |  |
| **Total** | | 747 (100) | |  | 1710 (100) |  | | | 2457 (100) | | |  | | |  | | | 2579 (100) | | |  | | | 2648 (100) | | |  | | | 5227 (100) | | |  | | |  | | | 3789 (100) | | |  | | | 3632 (100) | | | |  | | | 7421 (100) | | |  | | |  |
|  | |  | |  |  |  | | |  | | |  | | |  | | |  | | |  | | |  | | |  | | |  | | |  | | |  | | |  | | |  | | |  | | | |  | | |  | | |  | | |  |
| **Epilepsy** | |  | |  |  |  | | |  | | |  | | |  | | |  | | |  | | |  | | |  | | |  | | |  | | |  | | |  | | |  | | |  | | | |  | | |  | | |  | | |  |
| Yes | | 0 (0.0) | |  | 4 (0.2) |  | | | 4 (0.2) | | | 0.186 | | |  | | | 3 (0.1) | | |  | | | 3 (0.1) | | |  | | | 6 (0.1) | | |  | | |  | | | 1 (0.0) | | |  | | | 2 (0.1) | | | |  | | | 3 (0.0) | | | 0.539 | | |  |
| No | | 747 (100) | |  | 1706 (99.8) |  | | | 2453 (99.8) | | |  | | |  | | | 2576 (99.9) | | |  | | | 2645 (99.9) | | |  | | | 5221 (99.9) | | | 0.974 | | |  | | | 3788 (100) | | |  | | | 3630 (99.9) | | | |  | | | 7418 (100) | | |  | | |  |
| **Total** | | 747 (100) | |  | 1710 (100) |  | | | 2457 (100) | | |  | | |  | | | 2579 (100) | | |  | | | 2648 (100) | | |  | | | 5227 (100) | | |  | | |  | | | 3789 (100) | | |  | | | 3632 (100) | | | |  | | | 7421 (100) | | |  | | |  |
|  | |  | |  |  |  | | |  | | |  | | |  | | |  | | |  | | |  | | |  | | |  | | |  | | |  | | |  | | |  | | |  | | | |  | | |  | | |  | | |  |
| **Diabetes** | |  | |  |  |  | | |  | | |  | | |  | | |  | | |  | | |  | | |  | | |  | | |  | | |  | | |  | | |  | | |  | | | |  | | |  | | |  | | |  |
| Yes | | 21 (2.8) | |  | 41 (2.4) |  | | | 62 (2.5) | | | 0.548 | | |  | | | 79 (3.1) | | |  | | | 54 (2.0) | | |  | | | 133 (2.5) | | | 0.019 | | |  | | | 67 (1.8) | | |  | | | 63 (1.7) | | | |  | | | 130 (1.8) | | | 0.912 | | |  |
| No | | 726 (97.2) | |  | 1669 (97.6) |  | | | 2395 (97.5) | | |  | | |  | | | 2500 (96.9) | | |  | | | 2594 (98) | | |  | | | 5094 (97.5) | | |  | | |  | | | 3722 (98.2) | | |  | | | 3569 (98.3) | | | |  | | | 7291 (98.2) | | |  | | |  |
| **Total** | | 747 (100) | |  | 1710 (100) |  | | | 2457 (100) | | |  | | |  | | | 2579 (100) | | |  | | | 2648 (100) | | |  | | | 5227 (100) | | |  | | |  | | | 3789 (100) | | |  | | | 3632 (100) | | | |  | | | 7421 (100) | | |  | | |  |
|  | |  | |  |  |  | | |  | | |  | | |  | | |  | | |  | | |  | | |  | | |  | | |  | | |  | | |  | | |  | | |  | | | |  | | |  | | |  | | |  |
| **Jaundice** | |  | |  |  |  | | |  | | |  | | |  | | |  | | |  | | |  | | |  | | |  | | |  | | |  | | |  | | |  | | |  | | | |  | | |  | | |  | | |  |
| Yes | | 2 (0.3) | |  | 7 (0.4) |  | | | 9 (0.4) | | | 0.593 | | |  | | | 29 (1.1) | | |  | | | 32 (1.2) | | |  | | | 61 (1.2) | | | 0.777 | | |  | | | 24 (0.6) | | |  | | | 13 (0.4) | | | |  | | | 37 (0.5) | | | 0.092 | | |  |
| No | | 745 (99.7) | |  | 1703 (99.6) |  | | | 2448 (99.6) | | |  | | |  | | | 2550 (98.9) | | |  | | | 2616 (98.8) | | |  | | | 5166 (98.8) | | |  | | |  | | | 3765 (99.4) | | |  | | | 3619 (99.6) | | | |  | | | 7384 (99.5) | | |  | | |  |
| **Total** | | 747 (100) | |  | 1710 (100) |  | | | 2457 (100) | | |  | | |  | | | 2579 (100) | | |  | | | 2648 (100) | | |  | | | 5227 (100) | | |  | | |  | | | 3789 (100) | | |  | | | 3632 (100) | | | |  | | | 7421 (100) | | |  | | |  |
|  | |  | |  |  |  | | |  | | |  | | |  | | |  | | |  | | |  | | |  | | |  | | |  | | |  | | |  | | |  | | |  | | | |  | | |  | | |  | | |  |
| **HIV** | |  | |  |  |  | | |  | | |  | | |  | | |  | | |  | | |  | | |  | | |  | | |  | | |  | | |  | | |  | | |  | | | |  | | |  | | |  | | |  |
| Yes | | 1 (0.1) | |  | 3 (0.2) |  | | | 4 (0.2) | | | 0.814 | | |  | | | 10 (0.4) | | |  | | | 12 (0.5) | | |  | | | 22 (0.4) | | | 0.715 | | |  | | | 0 (0.0) | | |  | | | 3 (0.1) | | | |  | | | 3 (0.0) | | | 0.077 | | |  |
| No | | 746 (99.9) | |  | 1707 (99.8) |  | | | 2453 (99.8) | | |  | | |  | | | 2569 (99.6) | | |  | | | 2636 (99.5) | | |  | | | 5205 (99.6) | | |  | | |  | | | 3789 (100) | | |  | | | 3629 (99.9) | | | |  | | | 7418 (100) | | |  | | |  |
| **Total** | | 747 (100) | |  | 1710 (100) |  | | | 2457 (100) | | |  | | |  | | | 2579 (100) | | |  | | | 2648 (100) | | |  | | | 5227 (100) | | |  | | |  | | | 3789 (100) | | |  | | | 3632 (100) | | | |  | | | 7421 (100) | | |  | | |  |
|  | |  | |  |  |  | | |  | | |  | | |  | | |  | | |  | | |  | | |  | | |  | | |  | | |  | | |  | | |  | | |  | | | |  | | |  | | |  | | |  |
| **Other** | |  | |  |  |  | | |  | | |  | | |  | | |  | | |  | | |  | | |  | | |  | | |  | | |  | | |  | | |  | | |  | | | |  | | |  | | |  | | |  |
| Yes | | 34 (4.6) | |  | 116 (6.8) |  | | | 150 (6.1) | | | 0.034 | | |  | | | 164 (6.4) | | |  | | | 121 (4.6) | | |  | | | 285 (5.5) | | | 0.004 | | |  | | | 57 (1.5) | | |  | | | 62 (1.7) | | | |  | | | 119 (1.6) | | | 0.487 | | |  |
| No | | 713 (95.4) | |  | 1594 (93.2) |  | | | 2307 (93.9) | | |  | | |  | | | 2415 (93.6) | | |  | | | 2527 (95.4) | | |  | | | 4942 (94.5) | | |  | | |  | | | 3732 (98.5) | | |  | | | 3570 (98.3) | | | |  | | | 7302 (98.4) | | |  | | |  |
| **Total** | | 747 (100) | |  | 1710 (100) |  | | | 2457 (100) | | |  | | |  | | | 2579 (100) | | |  | | | 2648 (100) | | |  | | | 5227 (100) | | |  | | |  | | | 3789 (100) | | |  | | | 3632 (100) | | | |  | | | 7421 (100) | | |  | | |  |
|  | |  | |  |  |  | | |  | | |  | | |  | | |  | | |  | | |  | | |  | | |  | | |  | | |  | | |  | | |  | | |  | | | |  | | |  | | |  | | |  |

P-values were generated from chi-square and Fisher’s exact tests.

**Supplementary Table 6**: Patient Covid-19 information and their associated Seropositivity from June 2021 to June 2022

|  | **BURKINA FASO** | | | |  | **GHANA** | | | |  | **NIGERIA** | | | |
| --- | --- | --- | --- | --- | --- | --- | --- | --- | --- | --- | --- | --- | --- | --- |
|  | **IgG/IgM** | |  |  |  | **IgG/IgM** | |  |  |  | **IgG/IgM** | |  |  |
|  | **Positive** | **Negative** | **Total** | **P-value** |  | **Positive** | **Negative** | **Total** | **P-value** |  | **Positive** | **Negative** | **Total** | **P-value** |
|  | **n (%)** | **n (%)** | **n (%)** |  |  | **n (%)** | **n (%)** | **n (%)** |  |  | **n (%)** | **n (%)** | **n (%)** |  |
| **Hospital admission for more than a day in the past 12 months** |  |  |  |  |  |  |  |  |  |  |  |  |  |  |
| Yes, for illness during pregnancy | 5 (0.7) | 21 (1.2) | 26 (1.1) |  |  | 83 (1.5) | 57 (1.5) | 140 (1.5) |  |  | 71 (2.0) | 47 (1.2) | 118 (1.6) |  |
| Yes, for other illnesses | 152 (20.3) | 355 (20.8) | 507 (20.6) | 0.461 |  | 638 (11.3) | 330 (8.8) | 968 (10.3) |  |  | 162 (4.5) | 160 (4.2) | 322 (4.3) |  |
| Yes, for accident/injury | 13 (1.7) | 21 (1.2) | 34 (1.4) |  |  | 34 (0.6) | 43 (1.1) | 77 (0.8) | <0.001 |  | 28 (0.8) | 38 (1.0) | 66 (0.9) | 0.060 |
| No | 577 (77.2) | 1312 (76.8) | 1889 (76.9) |  |  | 4880 (86.6) | 3320 (88.5) | 8200 (87.4) |  |  | 3371 (92.8) | 3537 (93.5) | 6915 (93.2) |  |
| **Total** | 747 (100) | 1709 (100) | 2456 (100) |  |  | 5635 (100) | 3750 (100) | 9385 (100) |  |  | 3632 (100) | 3782 (100) | 7421 (100) |  |
|  |  |  |  |  |  |  |  |  |  |  |  |  |  |  |
| **Ever done a COVID-19 PCR test before?** |  |  |  |  |  |  |  |  |  |  |  |  |  |  |
| Yes | 65 (8.7) | 80 (4.7) | 145 (5.9) |  |  | 672 (11.9) | 373 (9.9) | 1045 (11.1) |  |  | 250 (6.6) | 177 (4.9) | 427 (5.8) |  |
| No | 682 (91.3) | 1630 (95.3) | 2312 (94.1) | <0.001 |  | 4963 (88.1) | 3377 (90.1) | 8340 (88.9) | 0.003 |  | 3539 (93.4) | 3455 (95.1) | 6994 (94.2) | 0.001 |
| **Total** | 747 (100) | 1710 (100) | 2457 (100) |  |  | 5635 (100) | 3750 (100) | 9385 (100) |  |  | 3789 (100) | 3632 (100) | 7421 (100) |  |
|  |  |  |  |  |  |  |  |  |  |  |  |  |  |  |
| **The outcome of the test result** |  |  |  |  |  |  |  |  |  |  |  |  |  |  |
| Positive | 10 (15.4) | 5 (6.3) | 15 (10.3) |  |  | 110 (16.4) | 37 (9.9) | 147 (14.1) |  |  | 26 (10.4) | 9 (5.1) | 35 (8.2) |  |
| Negative | 51 (78.5) | 71 (88.8) | 122 (84.1) | 0.180 |  | 509 (75.7) | 309 (82.8) | 818 (78.3) | 0.014 |  | 209 (83.6) | 154 (87.0) | 363 (85.0) | 0.118 |
| Don’t Know | 4 (6.2) | 4 (5.0) | 8 (5.5) |  |  | 53 (7.9) | 27 (7.2) | 80 (7.7) |  |  | 15 (6.0) | 14 (7.9) | 29 (6.8) |  |
| **Total** | 65 (100) | 80 (100) | 145 (100) |  |  | 672 (100) | 373 (100) | 1045 (100) |  |  | 250 (100) | 177 (100) | 427 (100) |  |
|  |  |  |  |  |  |  |  |  |  |  |  |  |  |  |
| **Time between most recent positive SARS-CoV-2 test and enrolment** |  |  |  |  |  |  |  |  |  |  |  |  |  |  |
| <1month | 7 (70.0) | 4 (80.0) | 11 (73.3) |  |  | 21 (19.1) | 5 (13.5) | 26 (17.7) |  |  | 3 (11.5) | 1 (11.1) | 4 (11.4) |  |
| 1-2 months | 1 (10.0) | 0 (0.0) | 1 (6.7) |  |  | 9 (8.2) | 6 (16.2) | 15 (10.2) |  |  | 5 (19.2) | 0 (0.0) | 5 (14.3) |  |
| 3-4 months | 0 (0.0) | 1 (20.0) | 1 (6.7) | 0.315 |  | 22 (20.0) | 10 (27.0) | 32 (21.8) | 0.353 |  | 4 (15.4) | 1 (11.1) | 5 (14.3) | 0.483 |
| >4 months | 2 (20.0) | 0 (0.0) | 2 (13.3) |  |  | 58 (52.7) | 16 (43.2) | 73 (50.3) |  |  | 14 (53.8) | 7 (77.8) | 21 (60.0) |  |
| **Total** | 10 (100) | 5 (100) | 15 (100) |  |  | 110 (100) | 37 (100) | 147 (100) |  |  | 26 (100) | 9 (100) | 35 (100) |  |
|  |  |  |  |  |  |  |  |  |  |  |  |  |  |  |
| **Time between most recent negative SARS-CoV-2 test and enrolment** |  |  |  |  |  |  |  |  |  |  |  |  |  |  |
| <1month | 8 (15.7) | 7 (9.9) | 15 (12.3) |  |  | 62 (12.2) | 47 (15.2) | 109 (13.3) |  |  | 15 (7.2) | 18 (11.7) | 33 (9.1) |  |
| 1-2 months | 6 (11.8) | 12 (16.9) | 18 (14.8) |  |  | 47 (9.2) | 30 (9.7) | 77 (9.4) |  |  | 28 (13.4) | 21 (13.6) | 49 (13.5) | 0.12 |
| 3-4 months | 4 (7.8) | 7 (9.9) | 11 (9.0) | 0.684 |  | 105 (20.6) | 77 (24.9) | 182 (22.2) | 0.166 |  | 34 (16.3) | 35 (22.7) | 69 (19) |  |
| >4 months | 33 (64.7) | 45 (63.4) | 78 (63.9) |  |  | 295 (58) | 155 (50.2) | 450 (55) |  |  | 132 (63.2) | 80 (51.9) | 212 (58.4) |  |
| **Total** | 51 (100) | 71 (100) | 122 (100) |  |  | 509 (100) | 309 (100) | 818 (100) |  |  | 209 (100) | 154 (100) | 363 (100) |  |
|  |  |  |  |  |  |  |  |  |  |  |  |  |  |  |

**Supplementary Table 7**: COVID-19 Vaccination uptake and reasons for not recommending vaccines

|  | **Total** | **Burkina Faso** |  | **Ghana** |  | **Nigeria** |
| --- | --- | --- | --- | --- | --- | --- |
|  | **n (%)** | **n (%)** |  | **n (%)** |  | **n (%)** |
| **COVID-19 vaccine status** |  |  |  |  |  |  |
| Yes, Dose 1 | 2906 (14.0) | 156 (6.3) |  | 2054 (20.6) |  | 696 (8.4) |
| Yes, Dose 1 & 2 | 2440 (11.8) | 38 (1.5) |  | 1945 (19.5) |  | 457 (5.5) |
| No | 15402 (74.2) | 2264 (92.1) |  | 5982 (59.9) |  | 7156 (86.1) |
| **Total** | **20748 (100)** | **2458 (100)** |  | **9981 (100)** |  | **8309 (100)** |
| **Vaccine types** |  |  |  |  |  |  |
| AstraZeneca | 3905 (73) | 55 (28.4) |  | 3095 (77.4) |  | 755 (65.5) |
| Sputnik V | 3 (0.1) | 0 (0.0) |  | 3 (0.1) |  | 0 (0.0) |
| Johnson &amp; Johnson | 547 (10.2) | 113 (58.2) |  | 432 (10.8) |  | 2 (0.2) |
| Pfizer | 207 (3.9) | 6 (3.1) |  | 149 (3.7) |  | 52 (4.5) |
| Moderna | 503 (9.4) | 11 (5.7) |  | 154 (3.9) |  | 338 (29.3) |
| Others (Sinopharm) | 7 (0.1) | 7 (3.6) |  | 0 (0) |  | 0 (0) |
| Don't Know | 174 (3.3) | 2 (1) |  | 166 (4.2) |  | 6 (0.5) |
| Total | 5346 (100) | 194 (100) |  | 3999 (100) |  | 1153 (100) |
| **Would you recommend someone take the COVID-19 vaccine?** |  |  |  |  |  |  |
| Yes | 17519 (84.4) | 1840 (74.9) |  | 8670 (86.9) |  | 7009 (84.4) |
| No | 3229 (15.6) | 618 (25.1) |  | 1311 (13.1) |  | 1300 (15.6) |
| Total | 20748 (100) | 2458 (100) |  | 9981 (100) |  | 8309 (100) |
| **What are your reasons for no recommendation?** |  |  |  |  |  |  |
| Long-distance to COVID-19 Vaccination Centre | 116 (3.6) | 33 (5.3) |  | 33 (2.5) |  | 50 (3.8) |
| Have little or no education on the COVID-19 vaccine | 691 (21.4) | 160 (25.9) |  | 229 (17.5) |  | 302 (23.2) |
| Long queue at the COVID-19 Vaccination Centre | 80 (2.5) | 25 (4.0) |  | 18 (1.4) |  | 37 (2.8) |
| The vaccine is said to change human DNA | 130 (4) | 40 (6.5) |  | 29 (2.2) |  | 61 (4.7) |
| It was meant to implant a chip into the human body | 146 (4.5) | 35 (5.7) |  | 13 (1.0) |  | 98 (7.5) |
| The vaccine will infect you with coronavirus | 201 (6.2) | 47 (7.6) |  | 59 (4.5) |  | 95 (7.3) |
| Fear of dying after taking the vaccine | 562 (17.4) | 66 (10.7) |  | 239 (18.2) |  | 257 (19.8) |
| I think it is bad | 698 (21.6) | 115 (18.6) |  | 265 (20.2) |  | 318 (24.5) |
| Others | 605 (18.7) | 97 (15.7) |  | 426 (32.5) |  | 82 (6.3) |
| **Total** | **3229 (100)** | **618 (100)** |  | **1311 (100)** |  | **1300 (100)** |

**Supplementary Table 8**: Association between participants’ vaccine recommendation and demographic variables (N = 20,748)

|  | |  |  |  | **Would you recommend someone take the COVID-19 vaccine?** | | | | | | | | |  |  |  | |  |  |  |
| --- | --- | --- | --- | --- | --- | --- | --- | --- | --- | --- | --- | --- | --- | --- | --- | --- | --- | --- | --- | --- |
|  | |  | **Burkina Faso** | | | |  | **Ghana** | | | | | |  | **Nigeria** | | | | | |
|  | **Total** | | **Yes** |  | **No** |  |  | **Total** | **Yes** |  | **No** | |  |  | **Total** | | **Yes** |  | **No** |  |
|  | | **No.** | **n (%)** |  | **n (%)** | **P- value** |  | **No.** | **n (%)** |  | **n (%)** | **P-value** | |  | **No.** | **n (%)** | |  | **n (%)** | **P-value** |
| **Age group (in years)** | |  |  |  |  |  |  |  |  |  |  | |  |  |  |  | |  |  |  |
| 4 -20 | | 276 | 208 (75.4) |  | 68 (24.6) |  |  | 1530 | 1243 (81.2) |  | 287 (18.8) | |  |  | 1251 | 987 (78.9) | |  | 264 (21.1) |  |
| 21-40 | | 1328 | 997 (75.1) |  | 331 (24.9) |  |  | 5057 | 4328 (85.6) |  | 729 (14.4) | |  |  | 4566 | 3806 (83.4) | |  | 760 (16.6) |  |
| 41-60 | | 668 | 501 (75.0) |  | 167 (25) | 0.055 |  | 2502 | 2279 (91.1) |  | 223 (8.9) | | < 0.001 |  | 1929 | 1713 (88.8) | |  | 216 (11.2) | <0.001 |
| 60+ | | 183 | 134 (73.2) |  | 49 (26.8) |  |  | 854 | 791 (92.6) |  | 63 (7.4) | |  |  | 554 | 496 (89.5) | |  | 58 (10.5) |  |
| Missing | | 3 | 0 (0.0) |  | 3 (100) |  |  | 38 | 29 (76.3) |  | 9 (23.7) | |  |  | 9 | 7 (77.8) | |  | 2 (22.2) |  |
| **Total** | | **2458** | **1840 (74.9)** |  | **618 (25.1)** |  |  | **9981** | **8670 (86.9)** |  | **1311 (13.1)** | |  |  | **8309** | **7009 (84.4)** | |  | **1300 (15.6)** |  |
| **Gender** | |  |  |  |  |  |  |  |  |  |  | |  |  |  |  | |  |  |  |
| Male | | 1129 | 879 (77.9) |  | 250 (22.1) |  |  | 3764 | 3275 (87) |  | 489 (13) | |  |  | 3252 | 2762 (84.9) | |  | 490 (15.1) |  |
| Female | | 1329 | 961 (72.3) |  | 368 (27.7) | 0.002 |  | 6217 | 5395 (86.8) |  | 822 (13.2) | | 0.741 |  | 5057 | 4247 (84) | |  | 810 (16.0) | 0.245 |
| **Total** | | **2458** | **1840 (74.9)** |  | **618 (25.1)** |  |  | **9981** | **8670 (86.9)** |  | **1311 (13.1)** | |  |  | **8309** | **7009 (84.4)** | |  | **1300 (15.6)** |  |
| **Highest educational level** | |  |  |  |  |  |  |  |  |  |  | |  |  |  |  | |  |  |  |
| None/Basic | | 1381 | 1038 (75.2) |  | 343 (24.8) |  |  | 1964 | 1723 (87.7) |  | 241 (12.3) | |  |  | 1733 | 1437 (82.9) | |  | 296 (17.1) |  |
| Secondary | | 725 | 547 (75.4) |  | 178 (24.6) | 0.581 |  | 5029 | 4330 (86.1) |  | 699 (13.9) | |  |  | 4304 | 3688 (85.7) | |  | 616 (14.3) |  |
| Undergraduate | | 228 | 162 (71.1) |  | 66 (28.9) |  |  | 2671 | 2342 (87.7) |  | 329 (12.3) | | 0.009 |  | 2068 | 1720 (83.2) | |  | 348 (16.8) |  |
| Postgraduate | | 124 | 93 (75.0) |  | 31 (25.0) |  |  | 220 | 183 (83.2) |  | 37 (16.8) | |  |  | 196 | 156 (79.6) | |  | 40 (20.4) | 0.004 |
| Missing | |  |  |  |  |  |  | 97 | 92 (94.8) |  | 5 (5.2) | |  |  | 8 | 8 (100) | |  | 0 (0.0) |  |
| **Total** | | **2458** | **1840 (74.9)** |  | **618 (25.1)** |  |  | **9981** | **8670 (86.9)** |  | **1311 (13.1)** | |  |  | **8309** | **7009 (84.4)** | |  | **1300 (15.6)** |  |
| **Monthly income** | |  |  |  |  |  |  |  |  |  |  | |  |  |  |  | |  |  |  |
| Low | | 1418 | 1023 (72.1) |  | 395 (27.9) |  |  | 7507 | 6495 (86.5) |  | 1012 (13.5) | |  |  | 5911 | 4962 (83.9) | |  | 949 (16.1) |  |
| Middle | | 728 | 560 (76.9) |  | 168 (23.1) |  |  | 2000 | 1790 (89.5) |  | 210 (10.5) | |  |  | 1102 | 952 (86.4) | |  | 150 (13.6) |  |
| High | | 311 | 257 (82.6) |  | 54 (17.4) | <0.001 |  | 125 | 108 (86.4) |  | 17 (13.6) | | <0.001 |  | 1291 | 1090 (84.4) | |  | 201 (15.6) | 0.162 |
| Missing | | 1 | 0 (0.0) |  | 1 (100) |  |  | 349 | 277 (79.4) |  | 72 (20.6) | |  |  | 5 | 5 (100) | |  | 0 (0.0) |  |
| **Total** | | **2458** | **1840 (74.9)** |  | **618 (25.1)** |  |  | **9981** | **8670 (86.9)** |  | **1311 (13.1)** | |  |  | **8309** | **7009 (84.4)** | |  | **1300 (15.6)** |  |
| **Employment type** | |  |  |  |  |  |  |  |  |  |  | |  |  |  |  | |  |  |  |
| Formal | | 317 | 237 (74.8) |  | 80 (25.2) |  |  | 1950 | 1739 (89.2) |  | 211 (10.8) | |  |  | 1443 | 1163 (80.6) | |  | 280 (19.4) |  |
| Informal | | 1611 | 1221 (75.8) |  | 390 (24.2) |  |  | 4697 | 4092 (87.1) |  | 605 (12.9) | |  |  | 4559 | 4067 (89.2) | |  | 492 (10.8) |  |
| Student | | 388 | 275 (70.9) |  | 113 (29.1) | 0.372 |  | 2086 | 1728 (82.8) |  | 358 (17.2) | | <0.001 |  | 1115 | 899 (80.6) | |  | 216 (19.4) | <0.001 |
| Unemployed/Retired | | 118 | 88 (74.6) |  | 30 (25.4) |  |  | 1096 | 963 (87.9) |  | 133 (12.1) | |  |  | 1183 | 874 (73.9) | |  | 309 (26.1) |  |
| Missing | | 24 | 19 (79.2) |  | 5 (20.8) |  |  | 152 | 148 (97.4) |  | 4 (2.6) | |  |  | 9 | 6 (66.7) | |  | 3 (33.3) |  |
| **Total** | | **2458** | **1840 (74.9)** |  | **618 (25.1)** |  |  | **9981** | **8670 (86.9)** |  | **1311 (13.1)** | |  |  | **8309** | **7009 (84.4)** | |  | **1300 (15.6)** |  |
