# Supplementary material for: Widespread SARS-CoV-2 exposure in West Africa: findings from a 20,748-participant multi-country seroepidemiology study
Source: Front Public Health. 2026 Oct 26;14:1945355. doi: 10.3389/fpubh.2026.1945355 (PMC13752853; doi:10.3389/fpubh.2026.1945355)
Supplement: Supplementary file 2 [file Supplementary_file_2.DOCX]

Supplementary Figures

**Supplementary Figure 1**: New cases of COVID-19 as reported by WHO on 07 June 2022. Graphs modified from <https://covid19.who.int/> . Blue borders indicate sampling periods from the different countries for this study. Yellow borders indicate previous seroprevalence study periods not reported in this study. This figure demonstrates temporal correspondence between our recruitment period and reported epidemic waves, not which variant infected individual participants.

**Supplementary Figure 2**: Map showing seroprevalence per recruitment site across three countries. Seroprevalence values are recruitment-site estimates and are not representative of the wider regions or countries. Colour scales differ between countries (Burkina Faso 0–33%, Ghana 31–70%, Nigeria 31–56%), so shading is not directly comparable across countries.

**Supplementary Figure 3:** Seropositivity among the unvaccinated across sampling periods. The labels correspond to the Delta-dominant (June–November 2021) and Omicron-dominant (December 2021–June 2022) sampling periods and indicate temporal correspondence with dominant circulating variants, not confirmed individual-level variant infection.

**Supplementary Figure 4**: Monthly breakdown of SARS-CoV-2 overall seropositivity (IgM and IgG) during the Delta- and Omicron-dominant waves in Ghana. The labels “Delta” and “Omicron” in the legend indicate temporal correspondence with dominant circulating waves and do not establish the infecting variant in individual participants.
